# Polysulfide Catalytic Materials for Fast‐Kinetic Metal–Sulfur Batteries: Principles and Active Centers

**DOI:** 10.1002/advs.202102217

**Published:** 2021-11-11

**Authors:** Menghao Cheng, Rui Yan, Zhao Yang, Xuefeng Tao, Tian Ma, Sujiao Cao, Fen Ran, Shuang Li, Wei Yang, Chong Cheng

**Affiliations:** ^1^ College of Polymer Science and Engineering State Key Laboratory of Polymer Materials Engineering Sichuan University Chengdu 610065 China; ^2^ State Key Laboratory of Advanced Processing and Recycling of Non‐Ferrous Metals Lanzhou University of Technology Lanzhou Gansu 730050 P. R. China; ^3^ Department of Chemistry Technische Universität Berlin Hardenbergstraße 40 Berlin 10623 Germany; ^4^ Department of Chemistry and Biochemistry Freie Universität Berlin Takustrasse 3 Berlin 14195 Germany

**Keywords:** catalytic materials and electrocatalysis, metal–sulfur batteries, polysulfide reduction/oxidation, redox kinetics, shuttle effects

## Abstract

Benefiting from the merits of low cost, ultrahigh‐energy densities, and environmentally friendliness, metal–sulfur batteries (M–S batteries) have drawn massive attention recently. However, their practical utilization is impeded by the shuttle effect and slow redox process of polysulfide. To solve these problems, enormous creative approaches have been employed to engineer new electrocatalytic materials to relieve the shuttle effect and promote the catalytic kinetics of polysulfides. In this review, recent advances on designing principles and active centers for polysulfide catalytic materials are systematically summarized. At first, the currently reported chemistries and mechanisms for the catalytic conversion of polysulfides are presented in detail. Subsequently, the rational design of polysulfide catalytic materials from catalytic polymers and frameworks to active sites loaded carbons for polysulfide catalysis to accelerate the reaction kinetics is comprehensively discussed. Current breakthroughs are highlighted and directions to guide future primary challenges, perspectives, and innovations are identified. Computational methods serve an ever‐increasing part in pushing forward the active center design. In summary, a cutting‐edge understanding to engineer different polysulfide catalysts is provided, and both experimental and theoretical guidance for optimizing future M–S batteries and many related battery systems are offered.

## Graphical abstract

The recent advances on designing principles and active centers for polysulfide catalytic materials toward M–S batteries are summarized. The reported chemistries and mechanisms, the rational design principles from catalytic polymers and frameworks to active sites loaded carbons, and primary challenges and perspectives are comprehensively discussed, which offers new understanding and guidance for engineering catalytic nanostructures in M–S batteries.

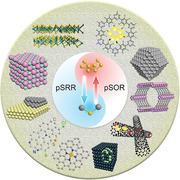

## 1 Introduction

Rechargeable lithium‐ion batteries (LIBs) have acted as a compelling character in alleviating increasingly serious energy dilemmas and the greenhouse effects.^[^ ^1^ ^]^ With the fast development of electric vehicles and portable electronic devices, LIBs gradually become insufficient to meet all the urgent demands on high energy density, low cost, safety, and sustainable performances.^[^ ^2^ ^]^ Recently, metal–sulfur batteries (M–S batteries, M = Li, Na, K) have been considered as the most promising candidates to succeed the conventional LIBs and satisfy the market demands thanks to their high specific energy density.^[^ ^3^ ^]^ Furthermore, sulfur is a reserve‐rich, inexpensive, and environmental‐friendly cathode material.^[^ ^4^ ^]^ Regardless of the merits mentioned above, several drawbacks have challenged the commercial applications, including I) the poor usage rate of sulfur because of the electrical insulation of elemental sulfur and its discharge product, II) the vast volume variation during the charge–discharge process chiefly deriving from the different densities of S and the reduced productions metal sulfides, and III) the notorious shuttle effects of diffusing polysulfide intermediates.^[^ ^5^ ^]^ The first two problems have been well solved by designing conductive and porous cathode materials.^[^ ^5^, ^6^ ^]^ However, as for the third problem, it is still a great challenge to suppress polysulfide shuttling effectively.

The sluggish kinetics of polysulfide reduction reaction (pSRR) and polysulfide oxidation reaction (pSOR), especially the pSRR process, result in flooding soluble polysulfides, which shuttle between the cathode and anode, thus inducing anode corrosion and severe self‐discharge.^[^ ^7^ ^]^ In the last few years, huge efforts have been devoted to addressing the above challenge,^[^ ^8^ ^]^ lots of strategies and materials have been proposed, including materials with physical and chemical adsorption to polysulfide or structure to entrap polysulfides, such as carbon materials,^[^ ^9^ ^]^ polymers, and ^[^ ^10^ ^]^ transition metal composites.^[^ ^11^ ^]^ Nevertheless, involving multielectron redox reactions and a series of sophisticated phase transformations, an inherently slow pSRR process cannot be accelerated effectively by mere adsorption from trapping materials.^[^ ^3^, ^7^, ^12^ ^]^

Therefore, the idea of electrocatalysis has been introduced into the M–S batteries to solve the kernel problem, especially catalyzing pSRR, which is to accelerate the sulfur species reduction, for instance, the conversion of Li_2_S_8_ → Li_2_S_6_ → Li_2_S_4_ → Li_2_S_2_/Li_2_S in a Li—S battery. A series of actively electrocatalytic materials have been discovered, which could achieve polysulfide's catalytic reduction/oxidation, thus not only promoting internal kinetics but also raising the capacity and rate performance of Li—S batteries.^[^ ^5b^ ^]^ Currently, diverse nanostructures with polysulfide catalytic capability have already sprung out. Duan and co‐workers have demonstrated that N,S‐co‐doped carbon aerogel exhibited high catalytic efficiency on polysulfide with good rate performance. This study also indicated that S_8_ was relatively easy to convert to polysulfide, while the conversion of soluble polysulfide to insoluble reduction products showed slower kinetics by activation energy tests.^[^ ^13^ ^]^ Meanwhile, the delicately designed conjugated porous polymers, metal–organic frameworks (MOFs), and inorganic compounds have also displayed good polysulfide catalytic capabilities.^[^ ^14^ ^]^ Most recently, profiting from the large surface area, good electroconductivity, high polarity, full accessibility, and unique electron configuration, abundant transition metal‐based single‐atom catalysts (SACs) have emerged as promising polysulfide catalytic materials.^[^ ^15^ ^]^ Although some early reviews have briefly described the chemistry and properties of polysulfide catalysis in Li—S batteries. The concepts of pSRR and pSOR established in recent years, the design principles and active centers of different polysulfide catalysts in M–S batteries, and the corresponding structure–function relationships have not been systematically reviewed. Thus, a cutting‐edge instructive review that summarizes the most advanced chemistries, mechanisms, and structure–function relationships is urgently needed, especially from the computational and theoretical aspects, to provide new inspiration and future direction to engineering the polysulfide catalytic centers in M–S batteries.

**Scheme 1:**
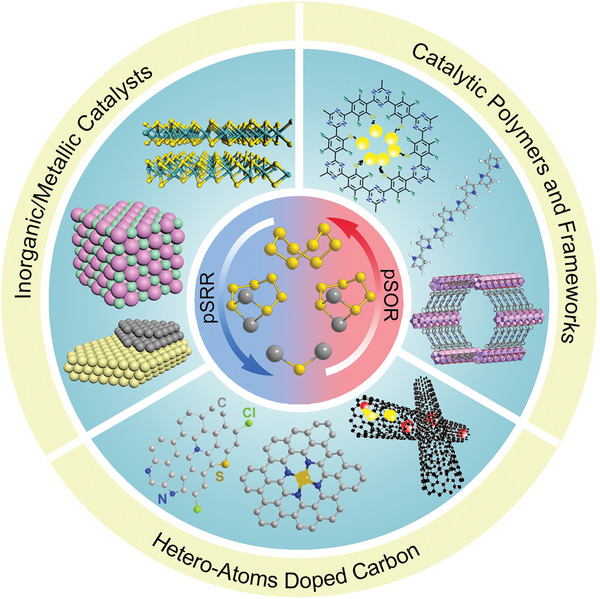
Illustrative image of diverse polysulfide catalytic materials for the redox reaction process in M–S batteries.

Here, this timely review highlights and systematically summarizes the most recent advances of designing principles and active centers for polysulfide catalytic materials toward the fast‐kinetic M–S batteries. First, the currently reported chemistry and mechanisms for the catalytic conversion of polysulfides are presented in detail. Subsequently, the rational design of polysulfide catalytic materials to accelerate the reaction kinetics in M–S batteries is comprehensively discussed, including the catalytic polymers and frameworks, inorganic/metallic catalysts heteroatoms doped carbon materials as shown in **Scheme** **1**. Notably, in these sections, we pay significant attention to the corresponding catalytic mechanisms and structure–function relationships, and computational methods serve an ever‐increasing part in pushing forward the active center design. Moreover, we have highlighted the current breakthroughs and identified the directions to guide future primary challenges, perspectives, and innovations. In summary, we provide a cutting‐edge understanding to engineer different polysulfide catalysts and offer both experimental and theoretical guidance for optimizing future high‐performance M–S batteries and many other related battery systems.

## 2 The Chemistries, Mechanisms, and Characterization Techniques of Polysulfide Catalytic Process

The chemistries and mechanisms of polysulfide catalytic conversion are slightly different in different M–S batteries. Taking the pSRR in the Li—S batteries as a representative example, a sulfur molecule (S_8_) reacts with Li^+^ to convert into high ordered polysulfides, then to several low ordered polysulfides, eventually to Li_2_S. The transformation of polysulfides from high ordered to low ordered is generally fast, but a mass of sulfur in the cathode lowers the reaction kinetics and enhances the polysulfide state residence time, leading to severe losses of active materials. The polysulfide detention time can be shortened by reaction kinetic enhancements, thus suppressing the dissolution.^[^ ^3a^ ^]^ In detail, the slow transition of polysulfides on the adsorptive substrates leads to a saturated state of polysulfides. Therefore, further polysulfide adsorption is blocked, and the proportion of dead sulfur increases.^[^ ^16^ ^]^ By contrast, the fast polysulfide conversion on catalysts leads to the nonsaturated state of polysulfides; thus, further adsorption of polysulfides can continue. Meanwhile, large extra driving power is necessary when Li_2_S_2_/Li_2_S turns to the soluble polysulfides in the charging process because of their ionic/electronic insulation and nonsoluble characters in the aprotic electrolyte, suggesting the sluggish oxidized process of Li_2_S_2_/Li_2_S and poor sulfur utilization.^[^ ^17^ ^]^ Therefore, improving the catalytic reaction kinetics to reduce the energy barriers from Li_2_S_2_/Li_2_S to polysulfides are supposed to utilize the sulfur effectively. Until now, various polysulfide catalytic materials have been reported for the accelerated catalytic kinetics.

### 2.1 Catalytic Process of pSRR

**Figure 1:**
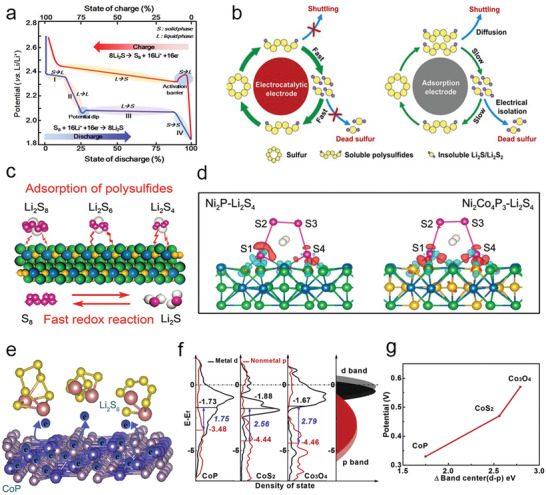
a) Scheme of working principles of Li—S battery. Reproduced with permission.^[^ ^3a^ ^]^ Copyright 2019, Wiley‐VCH. b) Advantages of catalytic materials compared with simple polar materials. Reproduced with permission.^[^ ^21^ ^]^ Copyright 2018, Elsevier. c) Model diagram of the interaction between Ni_2_Co_4_P_3_ nanowires and Li_2_S_6_. d) Optimized adsorption configuration for Li_2_S_4_ on Ni_2_P and Ni_2_Co_4_P_3_. c,d) Reproduced with permission.^[^ ^36^ ^]^ Copyright 2019, Wiley‐VCH. e) The schematic of Li_2_S_6_ battery on Co‐based compounds. f) Density of states analysis of anions and Co in different compounds, respectively. g) Scaling relation between the Δ band (d–p) center and Li—S redox potentials for different Co compounds. e‐g) Reproduced with permission.^[^ ^23^ ^]^ Copyright 2019, Elsevier.

As for a Li—S battery, the sluggish charge transport and soluble polysulfides shuttling are always ascribed to the faint affinity between polysulfides and conventional carbon mediators. Slow reduction of polysulfides will lead to their gathering in the electrolyte. Therefore, the electrochemical performance will be deteriorated due to the obstruction of the reaction pathway. Thus, the conversion of polysulfides is an important joint in the redox of sulfur.^[^ ^2c^ ^]^ Per sulfur atom can be reduced by two lithium atoms through two electrons conveying. While discharging, elemental sulfur is reduced to polysulfides and Li_2_S step‐by‐step (**Figure** **1**a): I) The first plateau, at about 2.4 V, relates to a two‐phase reaction, the conversion from solid S_8_ to dissolved high ordered polysulfides (Li_2_S*_x_*, 6 < *x* ≤ 8). II) The next ramp relates to the single‐phase reaction from high ordered polysulfides to dissolved low ordered polysulfides (Li_2_S*_x_*, 2 < *x* ≤ 6). As mentioned above, polysulfide dissolution chiefly happens in step (II); thus, enhancements of reaction kinetics are adopted to decrease polysulfide residence time, thereby suppressing the shuttle effect and alleviating the acute losses of polysulfide catalytic materials. III) The second plateau corresponds to the two‐phase reaction from low ordered polysulfides to solid Li_2_S_2_ is at about 2.1 V. IV) The last ramp relates to the single‐phase reaction from Li_2_S_2_ to Li_2_S.^[^ ^18^ ^]^ The electric potential decreasing at the start of the second plateau derives from the concentration polarization due to the obstacle of Li^+^ conveyance by the enhancement of electrolyte viscosity,^[^ ^3^, ^19^ ^]^ and the overpotential required by the crystallization of ionically/electrically insulating Li_2_S_2_.^[^ ^20^ ^]^ Low sulfur usage rate is caused by the inactive reaction kinetics in these steps. Since 75% of the discharge capacity (1254 mA h g^−1^) is contributed by step (III) and step (IV) (Li_2_S*_x_* →Li_2_S, *x* = 2, 4, 6), therefore, faster reaction kinetics is favorable for achieving the reversible cycle.^[^ ^3a^ ^]^

To reduce the shuttle effect and improve the sulfur usage rate under high sulfur content, some reports showed that the rapid conversion between lithium polysulfide and Li_2_S_2_/Li_2_S on the active sites of the electrocatalytic materials is faster than on the polar adsorbents by the only adsorption. As shown in Figure 1b, the catalytic materials not only adsorb polysulfide but also improve the redox reaction kinetics of adsorbing polysulfides by the convenient transfer of ions/electrons, which is better than the mere adsorption on polar adsorbents for polysulfides.^[^ ^21^ ^]^ Taking transition metal phosphides as an example, hollow polyhedron/CNT‐constrained nano‐CoP catalytic particles have been prepared to bind polysulfides and help to redox.^[^ ^24^ ^]^ Later, it has been found that the Co‐doped in Ni_2_Co_4_P_3_ could raise the d‐band of the metal site further,^[^ ^22^ ^]^ thus enhancing the interaction between catalysts and polysulfides and reducing the activation threshold. The theoretical calculation disclosed that the terminal S atoms were adsorbed to active sites by a strong Li—S bond (Figure 1c,d). Furthermore, the kinetic behaviors of Co‐based compounds have been systematically studied (Figure 1e–g), which showed that CoP had excellent polysulfide catalytic performance, mainly because the center of the p band in CoP raised significantly, decreasing the energy gap between the cobalt 3d and the anion 2p center at the Fermi level.^[^ ^23^ ^]^ Compared with other normal ions, P anion is softer, and the electron attraction is smaller, causing a rise in the energy of the bonding state and a decline in the energy gap between the bonding orbital and the antibonding orbital. The anions with more hybridization and contribution to the valence band electrons lead to a higher electron energy, propelling the electron exchange, and catalyzing interfacial S_6_^2−^/S^2−^ redox dynamics. Consequently, the moderate interaction between catalysts and polysulfides and the efficient charge transfer among them are beneficial to lower the reaction activation energy from soluble polysulfides to insoluble discharge products.

### 2.2 Catalytic Process of pSOR

The catalytic effects on M–S batteries can fall into catalytic reduction or oxidation processes. This section focuses on the catalysts in the oxidation of polysulfide. Like the reverse of a reduction reaction in a Li—S battery, one limitation could be the inherent insulation of Li_2_S_2_ and Li_2_S, causing poor ionic/electrical conductivity, which hampers the capacity and cycling life.^[^ ^25^ ^]^ The other issue is the irreversible precipitation of discharge productions on the cathode resulting in pore blocking and active material loss in the company of severe polarization, huge capacity degradation, and slow reaction kinetics.^[^ ^2c^ ^]^ In the face of such a serious situation, it is vital for realizing high reversible capacity and coulombic efficiency to understand and control the kinetics of the oxidation mechanism from insoluble sulfur species to soluble polysulfides during the charging process.

**Figure 2:**
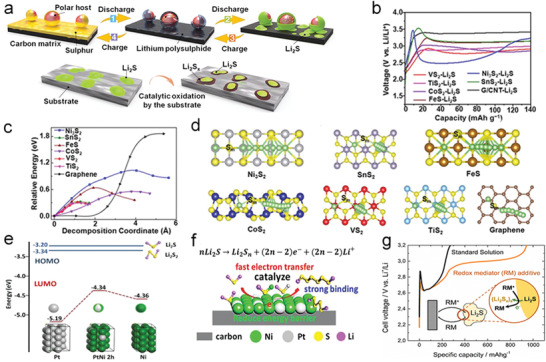
a) Scheme of the Li—S batteries sulfur conversion process and the oxidating process of Li_2_S with catalysts. b) Charge voltage profiles of electrodes in the first cycle. c) Energy distribution of Li_2_S clusters decomposition. d) Schematic illustration of Li_2_S decomposing on different sulfides. a‐d) Reproduced with permission.^[^ ^26^ ^]^ Copyright 2017, National Academy of Sciences. e) Diagram of the HOMO and LUMO energy levels. f) Schematic illustration of the Li_2_S catalytic oxidation mechanism. e,f) Reproduced with permission.^[^ ^27^ ^]^ Copyright 2019, Wiley‐VCH. g) Specific capacity comparison between standard solution and redox mediator additive. Reproduced with permission.^[^ ^28^ ^]^ Copyright 2014, American Chemical Society.

Tremendous efforts have been made to discover the mechanism of catalysis in the oxidation process. Most research works have underlined the conversion process in Li—S batteries, which includes four main steps (**Figure** **2**a). The key limits in deciding the energy obstacle for Li_2_S oxidation and polysulfide adsorption ability in Li—S batteries have been identified by systematically investigating a series of metal sulfides.^[^ ^26^ ^]^ Researchers speculated that the one Li_2_S molecule breaks up into one LiS cluster and one single Li^+^ ion at first. As shown in Figure 2b, the addition of CoS_2_, VS_2_, and TiS_2_ dramatically decreased the potential barrier to 3.01, 2.91, and 2.88 V, respectively. The binding process between S^2−^ in sulfides and separated Li^+^ is consistent with the decomposition process, which leads to that the sulfide materials can induce a low decomposition barrier compared with conventional carbon materials (Figure 2c,d).

Inspired by the excellent electrocatalytic activities of Pt and Ni in fuel cells, the Pt@Ni bimetallic material was reported to effectively reduce the energy barrier with simultaneously strong catalytic activity for the oxidation process of insoluble polysulfides to soluble polysulfides (Figure 2e,f).^[^ ^27^ ^]^ On account of a bifunctional mechanism, electron transferring from Ni to Pt was a propelling force for Ni‐activating Li_2_S decomposition by greatly promoting the transformation of Li—S—Li to Ni—S—Li, consequently liberating Li^+^ and electrons. The appearance of the intermediate state Ni—S—Li made the oxidization from Li_2_S to polysulfides easier. An abundance of activated —S—Li species was favorable to react with the Li_2_S, which binds to neighboring Pt sites, immediately freeing the Pt sites for the more catalytic reaction. Therefore, polysulfide catalytic materials with lithium‐philic or bifunctional catalytic sites are promising to transfer Li_2_S molecules into LiS clusters, thereby leading to easier oxidization of Li_2_S during the charge process.

### 2.3 Catalytic Reactions of Polysulfides with Redox Mediator (RM)

Besides, the RM is also important for the catalytic conversion of polysulfides by assisting both the reduction and oxidation processes. RM may be an electrolyte additive (Figure 2g) or simply the polysulfide,^[^ ^28^ ^]^ which aims to lower the free energy of phase decomposition and correspondingly diminish the initial barrier.^[^ ^29^ ^]^ Because of the insulating nature of S species,^[^ ^29^, ^30^ ^]^ during the charging process, most electrically isolated particles (M_2_S, M = Li, Na, K) can be oxidized into polysulfides at the localized interface of the electrode/electrolyte along with adequate charge transfer. Therefore, the M_2_S displays such a vast overpotential and a restricted reversible capacity that is inferior to the theoretical value.^[^ ^31^ ^]^ Furthermore, the dissolution–precipitation process produces insulating deposits of M_2_S, which significantly suppress the redox reaction due to the active interface passivated. Introducing RM to the cell systems signifies an effective strategy to ameliorate the limited performance.^[^ ^32^ ^]^ This approach depends on the electrochemical oxidation of RM in solution,^[^ ^30a^ ^]^ which can realize oxidizing the catalytic material on the entire surface of the particle. This extra charge transfer route in excess of the localized interface allows homogeneous and total oxidation of the electrode with a reduced overpotential.^[^ ^29^ ^]^

Recently, researchers have certified the feasibility of utilizing RM in polysulfide catalytic processes. Besides, during the discharge process, RM can devote itself to arresting polysulfides and speeding up Li_2_S nucleation in the polysulfide reduction process.^[^ ^33^ ^]^ Gerber et al. employed benzo[ghi] peryleneimide (BPI) as an RM to reduce polysulfides, which was able to transport electrons in short range.^[^ ^34^ ^]^ When charging to Li_2_S electrodes, proposing RM with higher redox potential than Li_2_S to the cell systems promotes the conversion of Li_2_S to polysulfides. However, the presolvated redox mediators may decrease efficiency when not immediately charging after assembly. Therefore, to address the challenge is utilizing a primarily resting RM, effective at low levels, segregating from the effect of electrolyte volume, and only activates at the first charge. Thus, it was then proposed that the oxidative decomposition of solid Li_3_PS_4_ can be leveraged as an RM generator that is electrochemical “switched on” for lowering the first charge overpotential of commercial Li_2_S.^[^ ^35^ ^]^

In general, the catalytic behaviors of pSRR and pSOR catalysts can be confirmed and explained by a series of theoretical calculations, such as the Li_2_S degradation energy, Li^+^ diffusion energy, binding energy, and reaction free energy calculations.^[^ ^15^, ^21^, ^36^ ^]^ Li_2_S molecules are usually degraded into LiS clusters and a single Li^+^ ion. In this process, a Li—S bond needs to be broken first, and then the single Li^+^ ion moves away from the S atom. Therefore, if the catalyst possesses a lithium‐philic site that can bind with free Li^+^ ions, the degradation process of Li_2_S will be promoted, which makes the oxidation of Li_2_S easier and faster.^[^ ^26^ ^]^ Besides, the Li^+^ ions diffusion energy can well reflect whether the catalyst can accelerate polysulfides’ redox reaction. Generally, faster Li^+^ ion diffusion facilitates the chemical reaction between S and Li at the catalyst surface.

Furthermore, the density of states and binding energy calculations between catalysts and polysulfides can reflect the chemical affinity between them and the inhibition ability of catalysts to the shuttle effects.^[^ ^37^ ^]^ Significantly, the reaction Gibbs free energy can judge the degree of difficulty in converting polysulfides, where catalysts usually lead to lower free energy for converting polysulfides, which thus accelerates the redox kinetic. Therefore, by combining a series of theoretical calculations, we can have a deep understanding of catalytic mechanisms during the pSRR and pSOR processes, thereby theoretically guiding the design of future catalysts.

### 2.4 Catalytic Reactions of Polysulfides in Na–S and K–S Batteries

Comparing with the thriving Li—S batteries, other types of M–S batteries, such as potassium–sulfur batteries (K–S batteries) and sodium–sulfur batteries (Na–S batteries) have some similar problems, generally about the volume variation and the migration of polysulfides. Like Li—S batteries, the generated polysulfide intermediates in Na–S and K–S systems are extremely soluble in electrolytes, leading to the terrible shuttle effect. Besides, the poor conductivity of sulfur species may lead to low electron transfer in the cathode. Therefore, the redox reaction kinetics and utilization of sulfur in Na–S batteries and K–S batteries get extremely depressed, thus generating large polarization.^[^ ^38^ ^]^ Furthermore, the K–S and Na–S batteries may differ from Li—S batteries in terms of the internal reaction pathways. For K–S batteries, the intermediate product from S_8_ to the final stable discharge product, K_2_S_3_, goes through several states, i.e., K_2_S_6_, K_2_S_5_, and K_2_S_4_.^[^ ^39^ ^]^ For Na–S batteries, there are several states, the Na_2_S_8_, Na_2_S_6_, Na_2_S_5_, Na_2_S_4_, and Na_2_S_3_ between S_8_ and the final discharge product (Na_2_S_2_ or Na_2_S).^[^ ^40^ ^]^ However, the phase transition process is similar, from insoluble sulfur to soluble high ordered polysulfides and finally to insoluble low ordered polysulfides. Similarly, the conversion between insoluble polysulfides is the most sluggish step. The critical point to rapidly catalyzing battery dynamics and reducing the shuttling effects remains to chemically anchor soluble polysulfides and accelerate the gaining and losing of electrons.^[^ ^41^ ^]^ Compared with Li—S battery catalysts, the development of these catalysts is still in the infant stage, the further identification of these polysulfide intermediates can guide the future theoretical calculation of catalysts and explore more efficient catalysts.

To solve the issues in M–S battery systems above, one solution is exploring appropriate cathode materials with suitable polarity, high conductivity, and sufficient exposed catalytic sites, as well as some effective structures, such as sheet, hollow, core–shell architectures. These solutions aim to construct functional adsorption sites and multiple open catalytic centers to strongly confine polysulfides and have a quick redox kinetic.

### 2.5 Characterization Techniques of Polysulfide Catalytic Process

**Figure 3:**
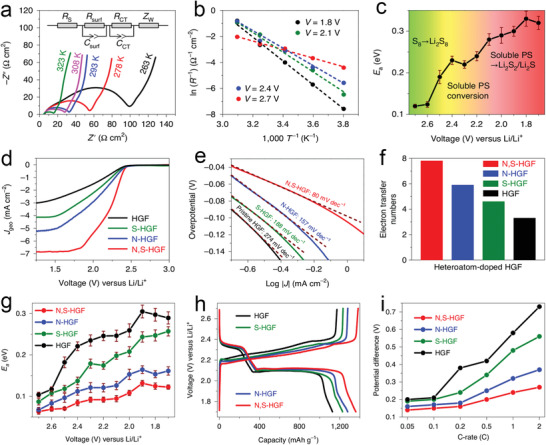
a) EIS measurements, b) Arrhenius plot, c) and activation energy profiles at various voltages based on Ketjen carbon black/sulfur composite cathode. d) LSV curves, e) Tafel plots, f) and electron transfer number comparison among heteroatom‐doped HGFs. g) Activation energies, h) charge/discharge curves, and i) the potential difference between the anodic and cathodic sweep in heteroatom‐doped HGFs. Reproduced with permission.^[^ ^13^ ^]^ Copyright 2020, Nature Publishing Group.

Lots of characterization techniques, such as activation energy test, rotary disk electrode test, density functional theory (DFT) calculation, and in situ tests, etc., have been applied to researching the dynamics of the polysulfides conversion reaction.^[^ ^42^ ^]^ Recently, the Duan group proposed that the reduction kinetics of pSRR at each step of transformation is related to the reaction activation energy *E*_a_ of the step, where the lower *E*_a_ leads to faster reduction kinetics.^[^ ^13^ ^]^ They obtained the activation energy at each test voltage (1.7–2.7 V) by fitting the charge transfer resistances of batteries measured at various temperatures into the Arrhenius equation. Specifically, the *E*_a_ was 0.12 eV at 2.70 V of S_8_→Li_2_S_8_, which increased to 0.24 eV at 2.40–2.10 V of Li_2_S_8_→Li_2_S_6_/Li_2_S_4_, and then reached a maximum value of 0.33 eV at 1.80 V of Li_2_S_4_→Li_2_S_2_/Li_2_S (**Figure** **3**a–c). These results indicated that S_8_ was relatively easy to convert to polysulfides, while the conversion of soluble polysulfides to insoluble products showed slower kinetics.

Apart from investigating the activation energy of various heteroatom‐doped porous graphene framework (HGF) as a catalyst, the Duan group also studied the reduction kinetics and reduction mechanism of pSRR. Interestingly, the linear sweep voltammetry (LSV) curves of pSRR showed similar characteristics to the oxygen reduction process when using the rotary disk electrode test, in which N, S‐HGF showed the highest half‐wave potential compared to single heteroatom‐doped or undoped HGF. Furthermore, the N, S‐HGF possessed the highest exchange current density, the lowest Taffel slope, and the largest electron transfer number, thereby exhibiting fast reaction kinetics and good electrochemical activity (Figure 3d–f). In the activation energy test, the *E*_a_ of different materials showed a noticeable difference, in which the *E*_a_ value decreased as HGF > S‐HGF > N‐HGF > N, S‐HGF. Meantime, the lower *E*_a_ of N, S‐HGF also resulted in the minimum polarization voltage gap, reflecting the lower overpotential and easier Li_2_S deposition of N, S co‐doped carbon (Figure 3g–i).

**Figure 4:**
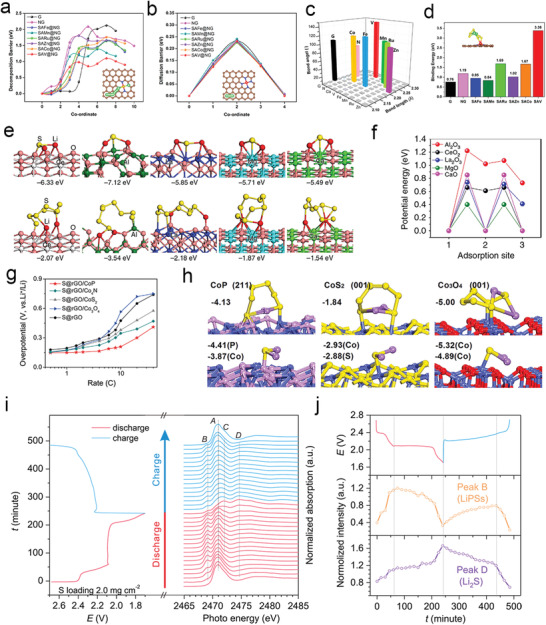
a) Decomposition barriers of Li_2_S, b) Li‐ion diffusion barriers, c) bond angle, and d) bond length of Li_2_S and binding energy of Li_2_S_6_ on different substrates. a‐d) Reproduced with permission.^[^ ^15b^ ^]^ Copyright 2020, American Chemical Society. e) The binding energy of metal oxides with Li_2_S and Li_2_S_8_, f) Li^+^ diffusion along with different adsorption sites on the oxide surface. e,f) Reproduced with permission.^[^ ^29^ ^]^ Copyright 2015, Nature Publishing Group. g) Overpotentials derived from the discharge/charge voltage plateaus and h) adsorption configurations and energies of Li_2_S_6_ and Li_2_S. g,h) Reproduced with permission.^[^ ^23^ ^]^ Copyright 2018, American Chemical Society. i) Evolution of S K‐edge XANES while cycling and j) the intensities of peak B. i,j) Reproduced with permission.^[^ ^36a^ ^]^ Copyright 2019, American Chemical Society.

DFT calculation is another powerful tool to predict materials with good properties. The Cui group has calculated six graphite‐based SACs with different metal centers by combining Li_2_S degradation energy,^[^ ^15b^ ^]^ Li^+^ diffusion energy, and binding energy in polysulfide catalysis. Notably, the Li^+^ diffusion energy was 0.23 eV for all SACs; however, the Li_2_S degradation energy showed a big difference: SAV@NG < SAMn@NG < SARu@NG < SAFe@NG < SACo@NG < SAZn@NG (**Figure** **4**a–c). Meanwhile, among all the catalysts, SAV@NG showed the strongest Li_2_S_6_ binding energy and the lowest free energy for polysulfide conversion (Figure 4d). Besides, Tao et al.^[^ ^29^ ^]^ used theoretical calculations of Li^+^ ion diffusion energy and binding energy to explore the catalytic behavior of a series of metal oxides (CeO_2_, Al_2_O_3_, La_2_O_3_, MgO, and CaO). Interestingly, the binding energy calculation proved that among all oxides, Al_2_O_3_ possessed the highest binding ability to Li_2_S_8_ and Li_2_S. However, the capacity decay during long cycles was the fastest (Figure 4e). Li^+^ ion diffusion calculations showed that the Li^+^ diffusion barrier on the surface of Al_2_O_3_ was the highest, which retarded the deposition and degradation of Li_2_S, thereby showing catalytic inertness (Figure 4f). Zhou et al. also calculated the binding energies of polysulfide with CoP, CoS_2_, Co_3_O_4_, and Co_4_N; they found that CoP showed moderate binding energies for Li_2_S_6_ and Li_2_S, thus showing the best Li_2_S diffusion kinetics and optimal electrochemical performances (Figure 4g,h).^[^ ^23^ ^]^

In situ/operando characterization techniques, including in situ X‐ray absorption spectroscopy, X‐ray diffraction, infrared spectra, and Raman spectra, have been extensively employed to get an in‐depth insight into the fundamental redox mechanism.^[^ ^41^, ^43^ ^]^ Banis and co‐workershave utilized the in situ X‐ray absorption near‐edge spectroscopy (XANES) to clarify particular mechanisms of Li—S batteries worked with an ether‐ or carbonate‐based electrolyte.^[^ ^44^ ^]^ Significantly, the in situ XANES disclosed a highly varying redox pathway for Li—S batteries with carbonate‐based electrolytes. Notably, there was no signal of the formation of soluble polysulfides during the charge and discharge process, owing to the instability of polysulfides in the carbonate‐based electrolyte. Furthermore, by using the in situ XANES characterization,^[^ ^36a^ ^]^ it was observed that an apparent intensification of peak D (concentration of Li_2_S) at the early stage of discharge (E > 2.1 V), suggesting the production of Li_2_S (Figure 4i,j). The early generation of Li_2_S during the discharge process revealed the accelerated electrochemical conversion during the phase change between the soluble polysulfides and insoluble Li_2_S_2_/Li_2_S. As a result, the advanced in situ/operando characterization techniques play crucial roles in probing the real‐time reaction process, thus guiding the future rational design of promising polysulfide catalytic materials.^[^ ^45^ ^]^ Therefore, advanced characterization techniques, such as activation energy test, rotary disk electrode test, DFT calculation, and in situ tests, etc., are desired to be adopted to deep understand the conversion mechanism of polysulfides anchored at catalytic sites.

As widely concerned materials, polysulfide catalytic materials, including various organic and inorganic electrocatalysts, exhibit up‐and‐coming advantages. As for the essential behaviors of sulfur cathodes, diverse types of organic and inorganic polysulfide catalytic materials play multilayered roles in serving as electrodes for M–S batteries.^[^ ^46^ ^]^ i) Conducting organic catalytic materials such as conducting polymers, covalent‐organic frameworks (COFs), and MOFs contribute to high S loading, powerful chemical affinity with polysulfides and convenient charge transfer. ii) Carbon‐free polar inorganic catalytic materials such as metal sulfides, metal nitrides, metal nanoparticles, and black phosphorus are general semiconducting, which helps achieve appropriate binding with polysulfides and fast electron movement between them, thus leading to accelerated redox kinetics. iii) Carbon materials with heteroatoms, metal–N*_x_*, metallic compounds loaded provide great promise to realize reduced internal resistance, high sulfur loading, and effective active centers to promote polysulfide redox kinetics.^[^ ^15b^ ^]^ Here, in the following sections, we will comprehensively review the structural design principles for efficient polysulfide catalysis and the rational designed catalytic active centers to accelerate the reaction kinetics in different types of polysulfide catalytic materials, including the catalytic polymers and frameworks, inorganic/metallic catalysts, and heteroatoms doped carbon materials, especially the most promising single‐atom catalysts and metallic compounds encapsulated porous carbon catalysts, offering guidance and inspiration for polysulfide catalytic materials in M–S batteries and promote their commercialization in energy‐related applications.

## 3 Catalytic Polymers and Frameworks for pSRR/pSOR in M–S Batteries

### 3.1 Conducting Polymers for Polysulfide Catalysis

Traditional conducting polymers, including polyaniline, polypyrrole, poly(3,4‐ethylenedioxythiophene), and poly(3,4‐ethylenedioxythiophene): poly(4‐styrene sulfonate) (PEDOT: PSS), are well known for their facilely synthetic processes, excellent elasticity, and good electroconductivity.^[^ ^47^ ^]^ Moreover, N‐doped groups and p‐conjugated structures always provide catalytic sites to immobilize and convert polysulfides in conducting polymers' backbone.^[^ ^48^ ^]^ Therefore, the conducting polymers have already been employed as sulfur hosts, separator modifier/functional interlayers, and cathode binders. Furthermore, conducting polymer‐based composites cathode, i.e., PEDOT:PSS/carbon black, can be easily constructed and prepared.^[^ ^49^ ^]^ With this cathode, it appeared excellent capacities and retention after 100 cycles mainly due to improving ionic and electronic conductivity.

Furthermore, when functionalized with groups like quinonoid imine,^[^ ^33^ ^]^ the conducting polymers can further chemically anchor polysulfide to promote redox reaction rapidly, which is promising for designing organic redox mediators in the M–S batteries. Therefore, from a long‐term perspective, it is worthwhile that conductive polymer should be functionalized with proper groups to accelerate polysulfide catalytic conversion. Recently, to confine polysulfides and solve safety problems, the strategy of the solid electrolyte has emerged for confining polysulfides by thermally cured composite polymer electrolyte as the barrier in Li—S batteries. For example, a kind of composite gel polymer electrolyte composed of poly‐(ethylene glycol) diacrylate, liquid electrolyte, and Li_6.4_La_3_Zr_1.4_Ta_0.6_O_12_ showed high ionic conductivity, good flame retardancy, and thermal stability. Besides, it can form a stable interface with the Li metal anode to inhibit the growth of lithium dendrites and avoid the occurrence of short circuits.^[^ ^50^ ^]^ Moreover, the iodine‐doped sulfurized polyacrylonitrile was prepared as sulfur hosts in Na–S and K–S batteries. The iodine doping could considerably enhance the conductivity of polymers by forming organic metal iodide.^[^ ^51^ ^]^

However, there are still some challenges remaining to be solved when using conducting polymers as polysulfide catalysts, such as 1) the relatively low conductivity of bare conducting polymers will result in insufficient electron transportation, integration of highly conductive materials is extremely necessary; 2) most of the reported organic conducting polymers exhibit low sulfur loading amount due to their inferior porosity compared to the porous carbon materials, thus combining porous materials with conducting polymers can be a promising strategy to engineer high‐performance sulfur cathodes; 3) the binding activity and catalytic conversion ability of these N‐doped conducting polymers are not sufficient compared to many polar metal compounds; therefore more efforts are needed to synthesize conducting polymers containing diverse heteroatoms to tune the binding and catalytic sites, such as B, O, S, P, and even metal ions or clusters.

### 3.2 COFs for Polysulfide Catalysis

**Figure 5:**
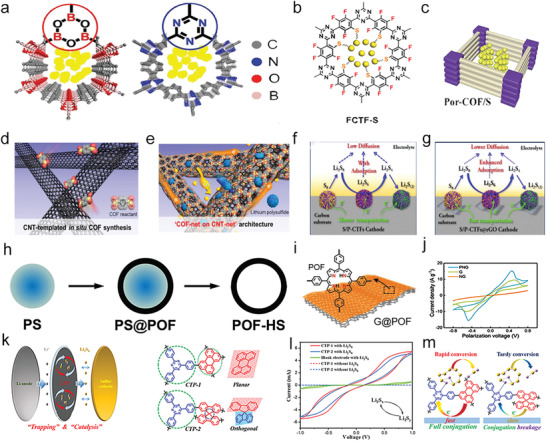
a) Chemical structure of N and B doped COFs. Reproduced with permission.^[^ ^55a^ ^]^ Copyright 2016, Wiley‐VCH. b) Chemical structure of FCTF‐S. Reproduced with permission.^[^ ^56^ ^]^ Copyright 2017, American Chemical Society. c) Schematic structures of porphyrin‐based POFs (Por‐POFs). Reproduced with permission.^[^ ^14a^ ^]^ Copyright 2016, Royal Society of Chemistry. d) Schematic synthesis of CNT‐templated in situ COF. e) Schematic structure of COF composite. d,e) Reproduced with permission.^[^ ^57^ ^]^ Copyright 2016, American Chemical Society. Schematic illustration of the discharge process in f) S/P‐CTF cathode, and g) S/P‐CTF@rGO cathode, rGO: reduced graphene oxide. f,g) Reproduced with permission.^[^ ^58^ ^]^ Copyright 2020, American Chemical Society. h) Schematic synthetic process of POF‐HS. Reproduced with permission.^[^ ^10c^ ^]^ Copyright 2018, Wiley‐VCH. i) Schematic illustration of G@POF. j) CV of symmetric cells of different electrodes. i,j) Reproduced with permission.^[^ ^59^ ^]^ Copyright 2018, Wiley‐VCH. k,m) Schematic diagram of the separators modified with triazine‐based polymers for trapping and catalysis of polysulfides in Li—S batteries. l) CV of symmetric cells. k‐m) Reproduced with permission.^[^ ^14b^ ^]^ Copyright 2016, Elsevier.

COFs, as new porous organic materials, have drawn significant attention since firstly reported by Yaghi's group in 2005.^[^ ^52^ ^]^ COFs with ordered structured and porous crystalline features can be delicately integrated by strong covalent bonds from basic organic building blocks with atomic accuracy.^[^ ^53^ ^]^ Because of the adjustable pore size and structure, permanent porosity, high specific surface area, and thermal stability, and low density, COFs have displayed promising potential in diverse fields, such as gas storage and catalysis.^[^ ^54^ ^]^ Recently, as shown in **Figure** **5**a–c, COFs have also been applied for the Li—S batteries using pristine boronate ester‐based COFs,^[^ ^55^ ^]^ and several N‐doped COFs.^[^ ^14^, ^56^ ^]^ Yoo et al. have firstly proposed a COF decorated mesoporous composite to trap polysulfides chemically as shown in Figure 5d,e.^[^ ^57^ ^]^ There was a critical point that the COFs with various pores served as polysulfides chemical traps, and the CNT hybrids with mesoporous played a role as ion‐conducting channels and electronic networks, which met the requirement for an ideal polysulfide trap. Besides the pristine COFs, a novel kind of porous phthalazinone‐based covalent triazine frameworks (P‐CTFs) with abundant N and O atoms have also been designed; the P‐CTFs can entrap the sulfur species by the strong chemical adsorption of polysulfides via the polar groups and accelerated electron transportation (Figure 5f,g).^[^ ^58^ ^]^

In reply to improving the high capacity, long cycling life, and superior rate performance of M–S batteries, macroporous and hollow structures are desired to be designed. Recently, to achieve this goal, the polystyrene microsphere was used as a template to design porphyrin organic framework (POF)‐based hollow sphere (POF‐HS) as shown in Figure 5h.^[^ ^10c^ ^]^ POF owns a well‐defined structure, simple synthetic methodology, and versatile functions. The N‐doped conjugated frameworks and versatile morphology of POF make it possible to create organic catalytic materials with precise nanostructures toward sulfur holding and catalytic conversion for Li—S batteries. Meanwhile, 2D porphyrin‐POF has also been synthesized using graphene as a substrate to contribute pyrrolic‐N or pyridinic‐N enriched nanosheets in Figure 5I,j,^[^ ^59^ ^]^ the exposed N species are capable of achieving the strong chemisorption of polysulfides to restrict their diffusion due to lithiophility, thus resulting in rapid kinetics for catalytic conversion of polysulfides. In addition to anchoring polysulfides with COFs, *π*‐conjugated donors in electron reservoirs exhibit different properties in catalyzing polysulfides. The test results showed that CTP‐1 presents a larger current density than CTP‐2 at a certain polarization voltage, showing faster reaction rates and kinetics of soluble polysulfides redox reactions (Figure 5l). It suggested that the conjugated triazinyl microporous polymers exhibit more rapid electron transport and polysulfide conversion than partially conjugated fractured polymers as shown in Figure 5k,m.^[^ ^14b^ ^]^

Besides the metal‐free COFs, Song's group combined the advantages of the COFs and metal atoms to synthesize the metal phthalocyanine COFs (MPc‐COFs, M = Ti, V, Mn, Cu, and Zn) as the cathodes in Li—S batteries.^[^ ^60^ ^]^ Through DFT calculations and experimental results of adsorption and catalysis of polysulfides, they suggested that the MPc‐COFs can facilitate the strong chemical adsorption capacity and catalysis for polysulfides than metal‐free phthalocyanine COF (HPc‐COF) due to the existence of Li–N and S–M interactions, especially TiPc‐COF and VPc‐COF. For the design of COFs in polysulfides catalysis, several main concepts have been adopted up to now: i) COFs are employed as porous organic substrates for chemical adsorption of polysulfides; ii) the realization of organo‐catalysts by edge functional groups on pore walls or enhanced conductivities; iii) anchoring single metal atoms within COFs pore is one of the most promising strategies for polysulfide catalysis;^[^ ^61^ ^]^ iv) another innovative solution is to directly prepare highly conductive conjugated COF materials to achieve efficient transmission of electrons. The synergy of in‐plane and interplane electron transmission will significantly enhance the transfer of electrons, thus shortening the distance between the electrons and active sites and enhancing M–S batteries' performance.

Pristine conductive COFs have great potential as sulfur hosts, but only preliminary results have been reported. Therefore, some challenges and reasonable solutions are advised as follows.1) Pristine COF materials may not be able to capture too much sulfur because only some sulfur molecules can enter micropores, so it is necessary to introduce a hierarchically porous structure to adsorb sulfur species and then utilize porous COFs to achieve the polysulfide catalytic conversion. 2) The application of COFs in M–S batteries has also been limited by their sluggish dynamic kinetics because of their poor conductivity. So integrating COFs with a conductive matrix and enhancing the electrical conductivity of COFs are good solutions toward promoting kinetics. 3) COFs own flexibility and stability, but their bulk forms always make their active sites inadequate exposure. Therefore, tuning the COF morphology with sheet‐like, core–shell, flower‐like, and 3D hierarchical structures is effective to maximize the exposed polar active sites, thus improving the electrochemical performance and alleviating the shuttle effect. 4) Some metal compounds that can bind and trap polysulfides, such as metal oxides, metal sulfides, and metal carbides, are needed to enhance battery performance. 5) Active heteroatoms can be introduced to functionalize the COFs molecules to enhance the chemical binding ability and catalytic activity to relieve the shuttle effects.

### 3.3 MOFs for Polysulfide Catalysis

Apart from COF‐based catalysts, the MOFs, as another type of organic frame materials, possessing uniform distributed nanopores and large specific areas, also provide promising opportunities to solve the critical problems in M–S batteries.^[^ ^5^, ^62^ ^]^ MOFs can be predesigned to own different hierarchical structures and rich polar/catalytic sites. The strong Lewis acid–base interaction between the open metal sites/clusters in the MOFs and polysulfides can suppress the shuttle effect. Additionally, compared with traditional inorganic porous or polar catalysts, MOFs exhibit superior catalytic performance owing to their uniformly distributed active sites and higher surface areas, and perform adequate binding ability for polysulfides, as well as efficient charge transfer.^[^ ^63^ ^]^ Based on these benefits of MOFs, in the past two years, researchers have investigated various MOFs and their modified materials for M–S battery systems.^[^ ^63^, ^64^ ^]^

**Figure 6:**
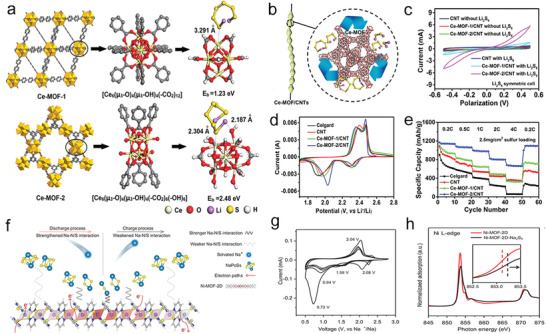
a) Illustration of active sites two MOFs. b) Schematic diagram of MOFs/CNT composite separation membrane structure for catalytic conversion of polysulfide. c) CV curves of different symmetrical cells at a scan rate of 50 mV s^−1^. d) CV curves of different cells at 0.1 mV s^−1^. e) Rate performance at different rates for the different separators. a‐e) Reproduced with permission.^[^ ^14c^ ^]^ Copyright 2019, American Chemical Society. f) Scheme of NaPSs confinement on 2D Ni‐MOF. g) CV curves of S/Ni‐MOF‐2D at a scan rate of 0.1 mV s^−1^. h) Ni L‐edge NEXAFS spectra of 2D Ni‐MOF (insert is electron transfer information). f‐h) Reproduced with permission.^[^ ^65^ ^]^ Copyright 2020, Wiley‐VCH.

Metal nodes/clusters containing coordinated unsaturated metal sites will lead to the generation of active centers, resulting in high catalytic performance in MOFs. It was reported that the cerium‐based MOF with more unsaturated coordination sites provides more active sites for rapid adsorption and catalytic conversion toward polysulfides (**Figure** **6**a,b).^[^ ^14c^ ^]^ The hexanuclear Ce (IV) clusters in Ce‐MOF‐1 coordinated with carboxyl groups had six more unsaturated coordination sites than the corresponding hexanuclear Ce (IV) clusters in Ce‐MOF‐2 surrounded by only six carboxyl groups. Therefore, compared with Ce‐MOF‐1, Ce‐MOF‐2 could provide more active sites for rapid adsorption and catalytic conversion toward polysulfides. Meanwhile, the CV curve of Ce‐MOF‐2/CNT showed two sharp redox peaks, the significant negative move of the oxidation peak and the significant positive move of the reduction peak, suggesting the reducing polarization and much better electrocatalysis (Figure 6c,d). Moreover, Ce‐MOF‐2/CNT exhibited the best rate performance and reversibility at different current densities (Figure 6e), which sufficiently proved that Ce‐MOF‐2/CNT held the function of catalyzing polysulfide conversion due to the accessible active centers.

Furthermore, besides the polysulfides, the local electronic state change of the metal in MOFs also has a significant enhancement effect in catalyzing the redox reactions of sodium polysulfides (NaPSs). As shown in Figure 6f, the Ni center with high redox capacity realized the transfer of electrons from 2D Ni‐MOF to NaPSs by strengthening Na–N/S interaction, which contributed to the sodiation process from Na_2_S_5_ to Na_2_S while discharging.^[^ ^65^ ^]^ During the charging process, the Na–N/S interaction weakened; meanwhile, electrons were transferred from NaPSs to 2D Ni‐MOF, which promoted the sodium removal process from Na_2_S to Na_2_S_5_. The Ni centers of S/Ni‐MOF showed dynamic electron states during charging and discharging processes, evoking tuning Na (sodium polysulfide)–N/S (2D Ni‐MOF) interaction, leading to fast redox kinetics of NaPSs (Figure 6g,h). Meanwhile, Ni electrons showed a decrease in the Ni L‐edge area, which also meant that electrons had been transferred from 2D Ni‐MOF to Na_2_S_5_ dynamically. Therefore, through mechanical stripping to change or rearrange the local electronic states of metal atoms, and dynamically adjusting the interaction between MOFs and polysulfides, the strong adsorption and rapid redox kinetics of polysulfides can be realized based on MOF carriers.

Consequently, the controllable chemical composition and high porosity of pristine organic framework materials should be emphasized due to the ability to capture polysulfides and tolerate volume expansions during cycling. Nevertheless, there are still some problems that need to be solved. 1) Most current reported MOFs are electrically insulating, leading to the requirements of additional conductive coating materials or experiencing high‐temperature carbonization.^[^ ^6^, ^66^ ^]^ Therefore, developing new conductive organic catalysts by introducing highly conjugated and conductive structures is highly desired, such as rising Cu[Ni(pdt)_2_] (pdt^2−^ = pyrazine‐2,3‐dithiolate), Cu‐BHT (BHT = benzenehexathiol), and Ni_3_(HITP)_2_ (HITP = 2,3,6,7,10,11‐hexaiminotriphenylene) etc.^[^ ^67^ ^]^ 2) High chemical and electrochemical stability against liquid electrolytes to ensure that these framework structures remain intact. Therefore, more stable and conductive MOFs should be designed and explored in M–S batteries. 3) Proper pore size that matches the diameter of an S_8_ molecule (0.69 nm) can be constructed to confine the sulfur and the corresponding reaction products inside MOFs. It should also be noted that a suitable porous structure and pore configuration are needed to facilitate fast Li^+^ transport dynamics and efficient sulfur confinement. 4) Sufficient exposed adsorption sites should be created to bind with polysulfides via both chemical interactions and framework confinements; meanwhile, secondary metal ions or metal clusters can be introduced into the MOF interiors to enhance the binding and catalytic ability. 5) Furthermore, the amount of surface defects also matters in cathode design, which can improve the intrinsic catalytic activity and polysulfide confinements. Therefore, it is necessary to explore the exact relationship between them for future MOF‐based cathodes.

## 4 Inorganic Catalysts for pSRR/pSOR in M–S Batteries

### 4.1 Inorganic Materials for Polysulfide Catalysis

Inorganic polysulfide catalysts, such as the carbon‐free metal compounds, metal nanoparticles/alloys, black phosphorus, MXene, etc., have possessed abundant accessible reactive sites and large polar surfaces,^[^ ^68^ ^]^ which are beneficial to achieve high sulfur loading, strong polysulfide immobilization, and fast redox kinetics in M–S batteries.

**Figure 7:**
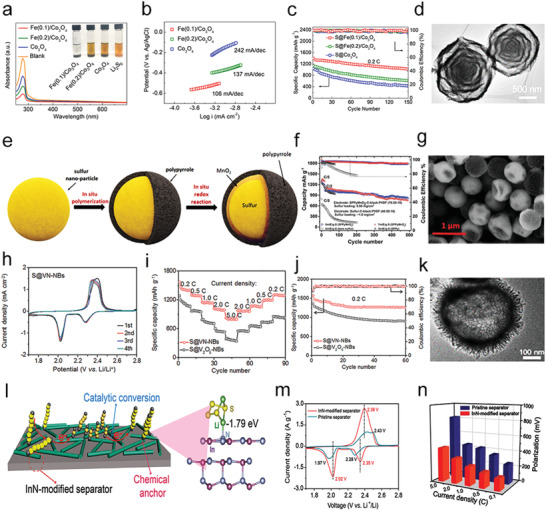
a) UV‐vis spectra and optical images of Fe(0.1)/Co_3_O_4_, Fe(0.2)/Co_3_O_4_, and Co_3_O_4_ adsorbing polysulfides. b) Tafel plots of Li_2_S oxidization on different substrates. c) Cycle performances at 0.2 C. d) TEM images of Fe/Co_3_O_4_. a‐d) Reproduced with permission.^[^ ^70a^ ^]^ Copyright 2020, American Chemical Society. e) Scheme of the synthesis process of SPPyMnO_2_ nanocomposite. f) Comparison of the efficiency and cycle performance of the bare sulfur, SPPy, and SPPyMnO_2_ electrodes. g) SEM image of the SPPyMnO_2_. e‐g) Reproduced with permission.^[^ ^71^ ^]^ Copyright 2018, Wiley‐VCH. h) CV curves of S@VN‐NBs cathode at 0.2 mV s^‐1^. i) Rate capabilities and j) cycling performances of S@VN‐NBs and S@V_2_O_5_‐NBs cathodes. k) TEM image of VN‐NBs. h‐k) Reproduced with permission.^[^ ^72^ ^]^ Copyright 2017, American Chemical Society. l) Scheme of the polysulfide conversion on InN. m) CV curves of different cells at 0.1 mV s^‐1^. n) Polarization potentials of Li‐S batteries at different current densities. l‐n) Reproduced with permission.^[^ ^7a^ ^]^ Copyright 2018, American Chemical Society.

Recently, a unique multishelled structure of Fe(0.1)/Co_3_O_4_ has been reported to provide multiple confinements for polysulfide trapping and alleviating the volume change during cycling (**Figure** **7**a,b). The Fe(0.1)/Co_3_O_4_ could act as an excellent electrocatalyst for the polysulfide conversions because of abundant oxygen vacancies from Fe doping. Generally, cation and anion defects could tune the electronic structure for good electrochemical properties, further influencing electron and ion transport properties.^[^ ^69^ ^]^ The oxygen defects theoretically arose out of low‐oxygen coordination, which was favorable toward attracting polysulfides, rapid charge transfer process, and enriched catalytic sites for polysulfide conversion, thus resulting in high utilization of sulfur and good cycling performances (Figure 7c,d).^[^ ^70^ ^]^ Therefore, with the combination of defect fabrication and surface/structure design, the recently reported Fe(0.1)/Co_3_O_4_ achieved highly improved states of electroconductivity, ion conductivity, and catalytic activity in Li—S batteries.

Moreover, to ensure a total encapsulation of sulfur, Chiang's group demonstrated a strategy to encapsulate sulfur nanoparticles by in situ reaction with manganese oxide particles and polypyrrole (Figure 7e,f).^[^ ^71^ ^]^ Here, to achieve an efficient encapsulation of S, they coated the S NPs with MnO_2_ particles as the interior shell since it was confirmed that soluble polysulfide species could be easily oxidized to thiosulfate groups by MnO_2_ particles.^[^ ^73^ ^]^ The thiosulfate groups forming on the surfaces of the MnO_2_ could facilitate the anchoring of long‐chain polysulfides via connecting them to form polythionates and therefore catalyzed their reduction to insoluble short‐chain polysulfides. Hence, the multilayer encapsulated binder‐free cathode admitted stable Li—S batteries with high‐load sulfur (Figure 7g,h).

In addition to metal oxides, metal nitrides have drawn lots of attraction in various catalytic fields because of their excellent conductivity, stability, and catalytic activity.^[^ ^74^ ^]^ With these features, metal nitrides have also been used as the cathode materials for M–S batteries. Many transition metal nitrides with excellent electrocatalytic properties have been applied to restrain the shuttle effect, such as vanadium nitride (VN),^[^ ^72^ ^]^ indium nitride (InN),^[^ ^7a^ ^]^ cobalt nitride,^[^ ^75^ ^]^ cubic nickel–iron nitride (Ni_3_FeN),^[^ ^68a^ ^]^ titanium nitride (TiN).^[^ ^68^, ^76^ ^]^

Jin's group created porous shell vanadium nitride nanobubbles (VN‐NBs) to host sulfur efficiently into the interior space (Figure 7I,j).^[^ ^72^ ^]^ Notably, with a highly porous and hollow structure, strong chemical adsorption and the catalytic ability for polysulfides, and high electrical conductivity for fast sulfur conversion, the cathodes based on sulfur encapsulated VN‐NBs could avoid the issues of low sulfur utilization and severe shuttle effect (Figure 7k,l). Furthermore, the polysulfide conversion kinetics could be efficiently promoted by an InN‐modified separator, which could be easily proved by the CV curves and polarization (Figure 7m–o).^[^ ^7a^ ^]^ InN with a narrow resolved bandgap possessed metal‐comparable properties, which helped the high electronic transferring on the surface during a redox reaction.

To obtain more active electrocatalytic performance, an extrinsic‐metal incorporating in situ etching strategy was proposed to activate Ni_3_N through Ni_3_FeN to serve as a vacancy‐sufficient catalyst. The Huang group verified that the catalytic activity always originated from the surface or subsurface defects and vacancies of a solid catalyst.^[^ ^68a^ ^]^ They transformed inactive Ni_3_N into a highly active cubic Ni_3_FeN phase after incorporating extrinsic iron to activate the inert Ni–N plane through polysulfide‐etching‐induced vacancies. Through electrochemical teats, it was demonstrated that the active center in Ni_3_FeN was mainly the vacancy that could strengthen the intermediate binding, lower the reaction barriers, and thus drive complete sulfur/polysulfide/Li_2_S conversion. Analogously, the inactive oxidation layers on TiN NPs were activated through the surface S doping using thermal treatment in H_2_S atmospheres, where the Ti—O bonds in the surface were partially replaced by Ti—S bonds. As a result, Ti—O bonds helped to adsorb polysulfides while the Ti—S bonds promoted electron transfer from the bulk TiN to the captured polysulfides on the surface due to their excellent electrocatalytic activity.^[^ ^68c^ ^]^

**Figure 8:**
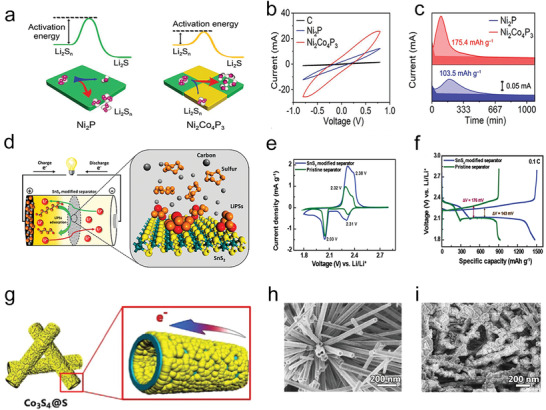
a) Activation energy difference of Li_2_S nucleation between using Ni_2_P and Ni_2_Co_4_P_3_ as catalysts. b) CV curves of the symmetric cells with different catalysts. c) CV curves of the symmetric cells with different catalysts. a‐c) Reproduced with permission.^[^ ^22^ ^]^ Copyright 2019, Wiley‐VCH. d) Schematic illustrations of Li—S battery with SnS_2_‐modified separator. e) CV curves of different cells at 0.05 mV s^−1^. f) The first cycle of discharge/charge curves with the different separators at 0.1C. d‐f) Reproduced with permission.^[^ ^78^ ^]^ Copyright 2019, Royal Society of Chemistry. g) Scheme of Co_3_S_4_@S nanotubes. SEM images of h) Co_3_S_4_ nanotubes and i) Co_3_S_4_/sulfur composite. g‐i) Reproduced with permission.^[^ ^79^ ^]^ Copyright 2017, Elsevier.

Metal phosphides also exhibit strong intrinsic affinity and strong chemical interaction with polysulfide, which can restrain the shuttle effect and catalyze the redox reaction.^[^ ^77^ ^]^ On the other hand, the good conductivity of metal phosphide is of great benefit to facilitating electron transportation.^[^ ^22^, ^77^ ^]^ Wang's group reported uniform Co–Fe phosphide nanocubes with pore architecture, which showed a high specific capacity and excellent cycling stability when used as the sulfur electrode.^[^ ^77a^ ^]^ More importantly, the DFT results showed that the strong interaction between polysulfide and Co–Fe–P led to the break of the Li_2_S_6_ molecular chain. Zhang and co‐workers designed Ni_2_Co_4_P_3_ nanowires as host materials for S cathodes, realizing 25 mg cm^−2^ ultrahigh sulfur loading with a capacity of 413 mA h g^−1^ (10 mA h cm^−2^) till 150 cycles.^[^ ^22^ ^]^ As shown in **Figure** **8**a–c, Ni_2_Co_4_P_3_ lowered more activation energy of conversion from Li_2_S*_n_* to Li_2_S, which could be attributed to the Co dopants in Ni_2_Co_4_P_3_ raising the d‐band of metal sites; after that, a redistribution of electron population resulted in the S—S bonds of Li_2_S*_n_* being easily broken.

Moreover, metal sulfides have recently been extensively studied for energy storage systems.^[^ ^80^ ^]^ On account of their superior affinity with sulfur species, metal sulfides have strong polysulfide adsorption and catalytic capacity. According to the Cui group calculation, the binding energies between metal sulfides and polysulfides are moderate; namely, the polysulfides bind with metal sulfides neither so strongly that they poison the active site nor too weakly to diffuse away.^[^ ^81^ ^]^ Therefore, lots of metal sulfides have been proposed to promote Li—S batteries' performance, such as Co_9_S_8_,^[^ ^82^ ^]^ CoS_2_,^[^ ^7b^ ^]^ VS_2_,^[^ ^83^ ^]^ FeS_2_,^84^ SnS_2_,^[^ ^78^ ^]^ VS_4_,^[^ ^85^ ^]^ Ni_3_S_2_,^[^ ^86^ ^]^ and ZnS.^[^ ^87^

Recently, the Kim group proposed an SnS_2_‐modified Celgard separator in an Li—S battery as shown in Figure 8d–f. The specially coated SnS_2_ modified separator could effectively trap polysulfides via robust chemical and physical interaction and guarantee Li ions’ fast diffusion. Besides, the SnS_2_ coating could serve as exceptional current collectors for promoting electron/ion transport, thereby improving sulfur utilization and efficiently accelerating the kinetic conversion of trapped polysulfides.^[^ ^78^ ^]^ Moreover, FeS_2_ has been used to restrain the shuttle effect in Li—S batteries and Na–S batteries as well.^[^ ^84^ ^]^ Interestingly, either in Li—S batteries or Na–S batteries, researchers have proved that the FeS_2_ component has undergone a chemical change and transformed into Li_2_FeS_2+_ *_n_* or Na*_x_* FeS_2_, which enhanced the adsorption and catalytic conversion of FeS_2_ to polysulfides. Zhang's group reported Co_3_S_4_@S nanotubes and investigated that the nanotubes performed better both on the polysulfide adsorption and catalytic kinetic enhancement compared with Co_3_S_4_ nanoparticles (Figure 8g–i) because the multifunctional nanotubes helped to form effective conductive networks.^[^ ^79^ ^]^ Therefore, inorganic polysulfide catalysts with desired nanostructures, such as core–shell, hollow, and multiple shelled hollow structures, could feature improved electron/ion transfer rate as well as easier accessible active sites.^[^ ^88^ ^]^

As a unique metal sulfide, the MoS_2_ belongs to 2D layered transition metal dichalcogenides, which have been widely explored recently due to their unique lamellar structure. Recently, Chen's group reported core–shell structured MoS_2_@S spherical cathodes for Li—S batteries.^[^ ^89^ ^]^ They found that the MoS_2_@S spheres constructed by 2D nanosheets exhibited superior mechanical suppression and chemical bonding toward polysulfides due to the unique core–shell nanostructure and the advantages of nanosheets. Notably, the nanocrystal structure of MoS_2_ includes edges and basal planes, and DFT calculations showed their interaction with Li_2_S in the series of Mo edge > S edge > terrace site.^[^ ^90^ ^]^ Moreover, the defect‐rich MoS_2_ nanosheets partially cracked in the inert terrace, thereby resulting in more active edge‐site exposure. Qiao group proposed that sulfur deficiency could lead to the larger charge densities of surrounding sulfur atoms on the MoS_2−_ *_x_* (001) surface than on MoS_2_ (001) based on calculation, thus enhancing the interaction of MoS_2−_ *_x_* with sulfur species.^[^ ^91^ ^]^ Therefore, it is of great expectation to fabricate defect‐rich MoS_2_ for advanced polysulfide catalysts.

**Figure 9:**
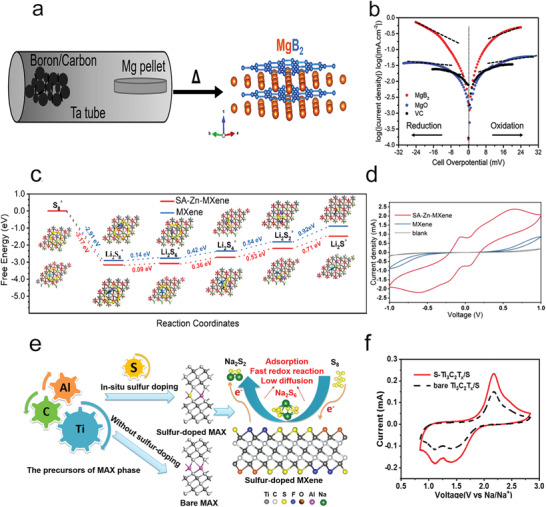
a) The schematic image of the synthesis process of MgB_2_ through a vapor–solid reaction. b) The Tafel plots of the Li_2_S_4_ solution redox on different host materials. a,b) Reproduced with permission.^[^ ^93^ ^]^ Copyright 2018, Elsevier. c) The Gibbs free energy of different lithium polysulfides on substrates. d) CV curves of different symmetric cells. c,d) Reproduced with permission.^[^ ^97^ ^]^ Copyright 2020, Wiley‐VCH. e) Scheme demonstrating the synthesis of sulfur‐doped MXene and the discharging process in sulfur‐doped MXene/S cathode. f) CV curves of Na–S cells with different cathodes in the second cycle. e,f) Reproduced with permission.^[^ ^98^ ^]^ Copyright 2019, American Chemical Society.

Additionally, many other emerged novel 2D materials have been proposed to employ in M–S batteries.^[^ ^92^ ^]^ Nazar's group developed a lightweight MgB_2_, which consisted of interleaved B and Mg layers, as a sulfur host to ensure both excellent electronic conduction and substantial polysulfides restriction (**Figure** **9**a,b).^[^ ^93^ ^]^ They demonstrated that both B‐ and Mg‐terminated surfaces could bond with the S*_x_* ^2−^ anions (not Li^+^) by first‐principles calculations, which promoted the electron conveying to the active S*_x_* ^2−^ ions. Black phosphorous (BP) has also displayed significant potential for the electrocatalysis of polysulfides because of its 2D features, low resistivity, high room‐temperature hole mobility, good bulk conductivity, fast Li^+^ ions diffusion constant, and high binding energies with sulfur.^[^ ^94^ ^]^ Recently, BP has been reported to catalyze the polysulfides redox reaction, the activity of which was ascribed to abundant active catalytic sites from edges.^[^ ^95^ ^]^ Moreover, Lin et al. demonstrated that the appearance of defects could promote the adsorption force between polysulfides and BP due to improved charge transfer by first‐principles calculation.^[^ ^96^ ^]^

MXenes, which were first reported in 2011 by the Gogotsi's group,^[^ ^99^ ^]^ gradually became a sort of promising electrode material because of the high conductivity, high lithium storage capacity, rapid diffusion of Li^+^ ions, and low operating voltage.^[^ ^5^, ^100^ ^]^ The early application of MXenes for energy storage was mainly limited to lithium‐ion batteries.^[^ ^101^ ^]^ Until more recently, MXenes have also been employed for M–S batteries.^[^ ^102^ ^]^ MXenes, the chemical formula of which should be M*_n_*_+1_X*_n_* T*_x_*, were a big category of 2D transition metal carbides, nitrides, and carbonitrides, including Ti_3_C_2_T*_x_*, V_2_CT*_x_*, Nb_2_CT*_x_*, and Ti_4_N_3_T*_x_* (T refers to functional groups on the surface, such as O, OH, S, Cl, F). However, most of the M–S batteries were engineered by Ti_3_C_2_T*_x_*‐based catalysts.^[^ ^103^ ^]^ For example, Qiu's group reported an MXene‐induced multifunctional collaborative interface, which possessed high conductivity and activity to adjust the kinetic behavior of polysulfide conversion.^[^ ^105^ ^]^ Similarly, Zhang and co‐workers found that the polar Ti_3_C_2_T*_x_* efficiently reacted with polysulfides and converted them into thiosulfate and a subsequent sulfate complex, which acted as a protective layer to suppress the polysulfides shuttle and to improve the utilization of sulfur.^[^ ^15^, ^105^ ^]^

Moreover, MXene with single atoms (SAs) doped could be a promising strategy to further improve its polysulfide catalytic effects (Figure 9c,d).^[^ ^97^ ^]^ The Yang group used single atom zinc implanted MXene layers (SA–Zn–MXene) as sulfur host, which could not only efficiently strengthen their interaction for polysulfides but also promote the conversion from Li_2_S_4_ to Li_2_S_2_ and Li_2_S. Furthermore, the SA–Zn–MXene layers could efficiently facilitate the nucleation of solid‐state Li_2_S_2_ and Li_2_S on their large exposed 2D surfaces. Besides, the Wang and Gogotsi groups have reported that surface‐functionalized MXene nanosheets prepared by in situ sulfur‐doping strategies were applied in RT–Na–S batteries as a cathode material (Figure 9e,f).^[^ ^98^ ^]^ Notably, the incorporation of sulfur terminations could significantly enhance the redox kinetics of Na–S batteries and restricted the diffusion of polysulfides, which leads to the good performance of RT Na–S batteries. Furthermore, the Gogotsi group has also demonstrated that S and O were the optimal options for Ti_3_C_2_ surface modification among various functional groups by DFT calculations.^[^ ^98^ ^]^ They proved that Ti_3_C_2_T_2_ (T = N, O, S) could adsorb Li_2_S_6_ and form S—T bonds to weaken the bonds in polysulfide and put forward that the priority order of kinetic properties in Li—S batteries, considering both catalysis and Li^+^ diffusion, was Ti_3_C_2_S_2_ > Ti_3_C_2_O_2_ > Ti_3_C_2_F_2_ > Ti_3_C_2_N_2_ > Ti_3_C_2_Cl_2_. Thus, designing MXene scaffold‐based catalysts or MXene with heteroatoms doped is believed to possess great expectation as an efficient catalyst for accelerating polysulfides redox reaction in M–S batteries.

Due to the polar nature of polysulfides, it has been reported that the strategies involving functional polar substrates as efficient sulfur hosts are very promising.^[^ ^106^ ^]^ The effects of polar metal oxides, metal sulfides, etc., have been discussed in the above sections. In a word, these polar materials exhibit enhanced properties due to their interfacial nature that can confine the polysulfides via polar–polar chemical interactions. However, there are still some challenges that need to be solved. 1) Most of these polar host materials own poor electroconductivity, leading to high charge transfer resistance and slow kinetics of polysulfides conversion, thus decreasing the sulfur utilization.^[^ ^107^ ^]^ In this regard, the strategy of developing conductive metal carbides or using conductive substrates to load polar catalysts may be a promising route. 2) Metal nitrides can facilitate the transport of ions and electrons of the polysulfide electrodes. However, the low porosity of metal nitrides results in low specific capacity, thus hindering electrochemical activity. Therefore, metal nitrides are always used in composites with other carbon‐based nanomaterials to physically and chemically confine polysulfides.

### 4.2 Metal Nanoparticles/Alloys for Polysulfide Catalysis

**Figure 10:**
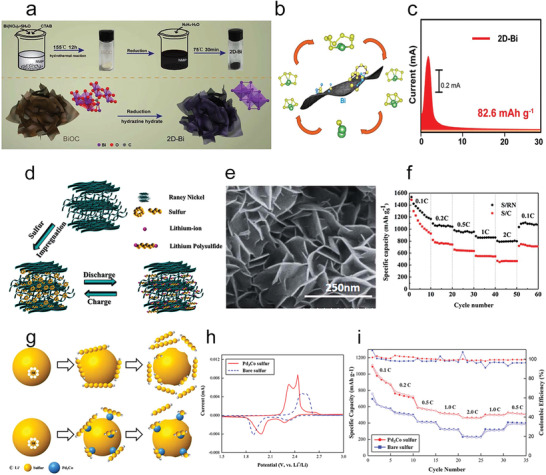
a) The preparation of the 2D‐Bi nanosheets. b) Schematic of the interior conversions of Li_2_S*_x_* on 2D‐Bi nanosheets. c) Chronoamperometry curves at 2.08 V. a‐c) Reproduced with permission.^[^ ^109^ ^]^ Copyright 2020, Royal Society of Chemistry. d) Scheme of sulfur impregnation into RN and restriction of polysulfides during cycling. e) SEM image of RN. f) The discharge capacities of the different cathodes. d‐f) Reproduced with permission.^[^ ^111^ ^]^ Copyright 2017, Royal Society of Chemistry. g) Scheme of the discharge process in bare sulfur and Pd_3_Co electrodes. h) CV plots of Pd_3_Co and bare sulfur electrodes at 100 mV s^−1^ after the first cycle. i) Rate capacities of cells with Pd_3_Co and bare sulfur electrodes at different rates. g‐i) Reproduced with permission.^[^ ^112^ ^]^ Copyright 2016, The Electrochemical Society.

Inspired by that metals are active catalysts for oxygen reactions, researchers have applied metallic materials into M–S batteries.^[^ ^3a^ ^]^ Notably, the electrochemical stability of metals should be taken into account because of the demand for electrode stability and the possible formation of metal compounds due to side reactions during battery operation.^[^ ^108^ ^]^ Li's group synthesized ultrathin 2D‐Bi nanosheets from precursor Bi_2_O_2_CO_3_ (BiOC) and employed them as effective multifunctional catalysts for polysulfide redox (**Figure** **10**a–c).^[^ ^109^ ^]^ The 2D Bi has been confirmed to be an excellent cathode material to effectively catalyze the solid–liquid conversion and stimulate the forward–reverse polysulfide redox reactions. Besides, Ni, Pd, and Pt were also stable metals with fantastic catalytic capacities.^[^ ^110^ ^]^ The Ni‐based sponge‐like porous material, RANEY nickel (RN), has been prepared to act as a new immobilizer to host sulfur (Figure 10d–f).^[^ ^111^ ^]^ As shown, the S/RN cathode had exhibited good rate performance, which should be attributed to the excellent electroconductivity of RN. The RN immobilizer formed a Ni chemical bond with sulfur grains, thus acting as both physical and chemical adsorbers. The strong chemical bond with sulfur species and excellent electronic conductivity made it easy to promote the kinetics during catalytic conversion of polysulfides. The palladium–cobalt (Pd_3_Co) alloy nanoparticles can be utilized as a cathode additive with efficient redox reaction kinetics for Li—S batteries without any reformed structure (Figure 10g).^[^ ^112^ ^]^ The CV curve and rate capacities indicated the superior electrical conductivity and stable lithium polysulfide conversion reaction kinetics of Pd_3_Co (Figure 10h,i). These results validate the importance of metal catalysts in M–S batteries, which can considerably diminish the shuttle effect because of their active catalysis for polysulfides redox reactions.

Currently, there are only very limited reports showing that metals and alloys can be used to catalyze the electrochemical conversion of polysulfide. Since the polysulfide adsorption on metal surfaces is the first step during the electrocatalytic process, the formation of metal compounds, for example, metal sulfides, are inevitable during the charge and discharge processes, which may significantly influence the surface redox electrochemistry and battery performance. Besides, it is also a challenge to maximize the catalytic sites of the metal nanoparticle‐loaded catalysts compared to the single‐atom catalysts. By introducing the carbon or other porous substrate may change the porosity, enhance the density of catalytic sites, and modify the metal's valence band center, thus tuning the binding ability and catalytic effects.

## 5 Carbon Supported pSRR/pSOR Catalysts for M–S Batteries

Carbon materials, including traditional disorder carbons, recent reported carbon nanotubes, and various porous nanocarbons, are greatly needed due to their low price, excellent conductivity, high specific surface areas, and satisfactory physical/chemical strength. These features are all contributing to constructing high‐performance polysulfide catalysts. Another special characteristic of these carbon materials should be the ability to be decorated with various heteroatoms, which can tune the electronic structures of carbon skeletons.^[^ ^98^ ^]^ These modification strategies have been frequently used to enhance the catalytic activities of pristine carbons in M–S batteries.^[^ ^113^ ^]^ Two mainstream pathways for creating catalytic carbons are proposed. i) Doping single or multiple heteroatoms into carbon frameworks to increase their polarity and produce active carbon atoms.^[^ ^114^ ^]^ ii) Introducing metal‐based catalytic centers, such as metal NPs, single metal atoms, and metallic compounds to achieve strong polysulfide confinement and fast redox kinetic.^[^ ^21^, ^115^ ^]^

### 5.1 Metal‐Free Carbons for Polysulfide Catalysis

**Figure 11:**
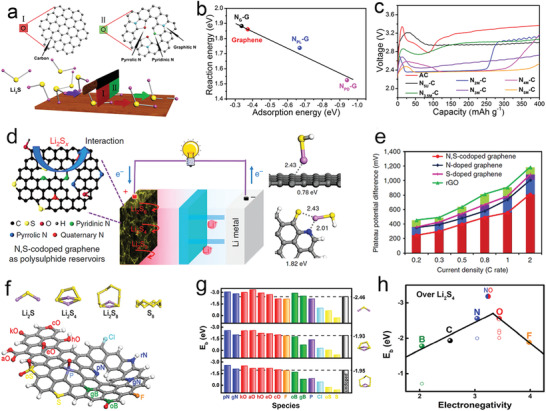
a) Evolution of the solid Li_2_S to ionized LiS^−^ and Li^+^ ions based on N‐doped carbon. b) The reaction energy and c) activation energy of various N‐contained structures. a‐c) Reproduced with permission.^[^ ^117^ ^]^ Copyright 2018, Elsevier. d) The interaction of polysulfides with N, S‐co‐doped graphene electrode. e) Different charge and discharge plateaus are based on N or/and S doped graphene and pristine graphene. d,e) Reproduced with permission.^[^ ^118^ ^]^ Copyright 2015, Nature Publishing Group. f) Heteroatom doped nanocarbon materials, and g) their binding energy *E*_b_ (eV) toward polysulfides. h) The relationship between *E*_b_ (toward Li_2_S_4_) and the special electronegativity of dopants. f‐h) Reproduced with permission.^[^ ^119^ ^]^ Copyright 2016, Wiley‐VCH.

Due to the facile preparation process, chemical stability, tunable structure, particular electronic configuration, and facile preparation, the heteroatoms doped carbons are extremely promising catalysts for polysulfides. Lately, heteroatoms doped carbons have been successfully used to entrap the polysulfides because of their strong chemical interaction between active sites and polysulfides.^[^ ^116^ ^]^ Besides, the N‐doped carbon could act like a sharp knife to break the binding between Li and S to decrease the activation barriers. The dissociation energies of Li_2_S in delithiation kinetics on the surface of different N‐doped carbon were calculated by DFT simulation (**Figure** **11**a–c).^[^ ^117^ ^]^ Pyrrolic and pyridinic N‐doped carbons were both beneficial in decreasing the decomposition energy of Li_2_S. More importantly, the pyridinic N showed the optimum doping structure of carbon for Li—S batteries, not only holding the strongest adsorption toward polysulfides but providing decomposition of Li_2_S with the least activation energy.

It has been reported that nitrogen and sulfur co‐doped carbons could considerably promote the catalytic ability for an oxygen reduction process.^[^ ^114^ ^]^ Motivated by the above work, the Manthiram group has demonstrated an N, S‐co‐doped carbon to host sulfur,^[^ ^118^ ^]^ which could significantly improve electroconductivity, promote affinity for polysulfides, and support high‐rate kinetics. Electrochemical tests demonstrated that the discharge/charge profiles of the N, S co‐doped graphene cathode had an apparent higher discharge plateau and a longer plateau compared with the rGO and single S/N doped graphene (Figure 11d,e). Those long and flat plateaus with smaller polarization could be well kept even at 0.3–2C rates, suggesting better redox reaction kinetics. Significantly, the electrochemical performance of heteroatoms (B, N, O, F, P, S, Cl) doped carbon electrodes for polysulfide catalysis has been systematically studied (Figure 11f–h).^[^ ^119^ ^]^ DFT results revealed that N and O dopants could significantly improve the interaction between carbon and polysulfides due to their high electronegativity and a suitable radius to match the Li atom.

**Figure 12:**
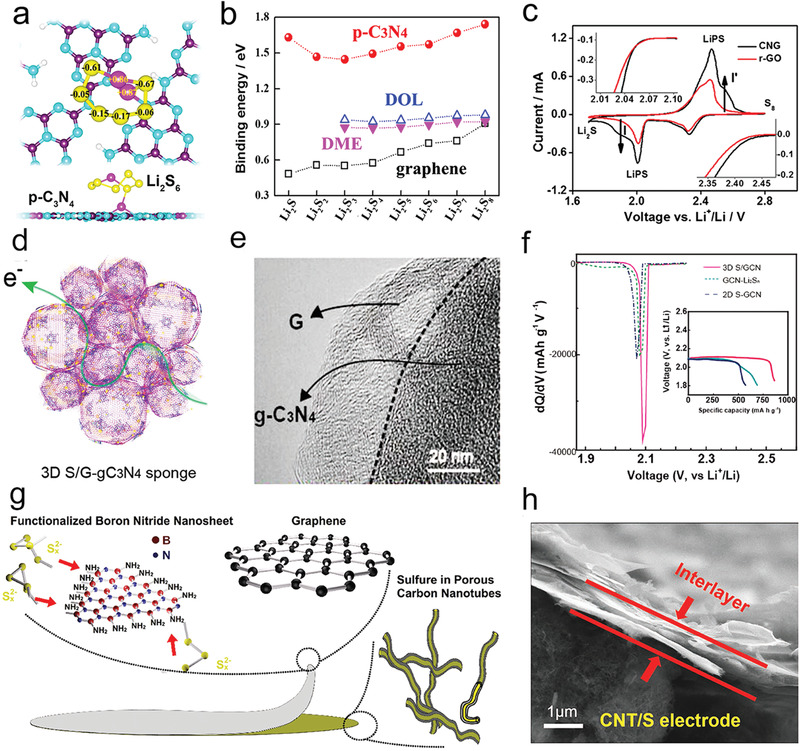
a) The interaction of polysulfides with p‐C_3_N_4_. b) The binding energy of LiPSs with DOL/DME and LiPSs on the substrate surface. c) CV curve differentiation that is based on the CNG and r‐GO electrode in Li—S batteries. a‐c) Reproduced with permission.^[^ ^120^ ^]^ Copyright 2016, American Chemical Society. d) The hybrid structure and e) TEM image of S/GCN sponge. f) d*Q*/d*V* curves of S/GCN, GCN‐Li_2_S*_n_*, and S‐GCN electrodes. d‐f) Reproduced with permission.^[^ ^121^ ^]^ Copyright 2018, Wiley‐VCH. g) Schematic and h) SEM image of CNT/S electrode with an FBN/G interlayer in Li—S cell. g,h) Reproduced with permission.^[^ ^123^ ^]^ Copyright 2017, Wiley‐VCH.

Besides, the heteroatoms doped carbons, carbon nitride (C_3_N_4_) and boron nitride (BN), are also promising catalysts for M–S batteries. An interaction induced by static between p‐C_3_N_4_ and polysulfides has been reported to promote the kinetics of polysulfides redox reaction,^[^ ^120^ ^]^ thus leading to a considerably improved battery performance (**Figure** **12**a–c). Simultaneously, DFT calculations disclosed that this special kinetic improvement of the polysulfides redox derived from the strong adsorption for polysulfides from p‐C_3_N_4_ and resultant polysulfide molecular structure distortion. Moreover, a kind of 3D porous graphene@g‐C_3_N_4_ (GCN) composite sponge was synthesized as an electrode for Li—S batteries.^[^ ^121^ ^]^ Here, the abundant N‐sites in GCN macropores provided lots of adhesive sites for polysulfides, achieving a physicochemical dual‐confinement for polysulfides. Meanwhile, the 3D inflexible graphene linkage could boost fast electron/ion delivery and retain structure integrity, thus guaranteeing quick redox kinetics and long‐range cycling stability (Figure 12d–f). Besides, as isoelectronic species, BN and graphene possessed similar features in a configuration. The graphene‐supported BN nanosheet hybrids thereby exhibited an alternative surface electronic structure resulting in a great different adsorptional characteristic. Meanwhile, the graphene/BN hybrids with high catalytic activity for polysulfides conversion in a Li—S battery within a broad range of temperatures has been developed.^[^ ^122^ ^]^ Recently, a functionalized BN nanosheets/graphene interlayer for Li—S batteries has also been reported as shown in Figure 12g,h. Due to the ultralight and thin interlayer, the cathode exhibited remarkably improved cycle stability.^[^ ^123^ ^]^

Despite the excellent performance of metal‐free carbon for M–S batteries, some key points remain to be explored for further development. 1) Traditional nanoporous carbon materials present certain drawbacks, such as disordered structures and nonuniformed sizes, which may lead to insufficient confinement and durability in M–S batteries.^[^ ^124^ ^]^ Hence, nanostructured carbons with precise control of the size, shape, composition, and structure are desired, in which MOF‐derived carbons are good choices. 2) Since the fabricated heteroatom‐doped carbon mainly contains N, S, O, and P groups, the interfacial polarity is relatively low; introducing more polar catalytic metal or metal clusters is needed. 3) As for heteroatom‐doped metal‐free carbon, the exact reaction mechanism is still not fully understood, and the precise dopant locations and structure of heteroatoms in catalysts are still unclear. Therefore, controllable synthetic strategies, such as atomic layer deposition and controlled precursor synthesis, should be developed to fully disclose the catalytic mechanisms. Moreover, advanced characterizations are required, such as atomic electron microscopy, operando spectroscopy technology, etc., to present geometric and electronic information quantitatively.

### 5.2 Single‐Atoms Doped Carbons for Polysulfide Catalysis

Single‐atoms doped carbon catalysts, also named SACs with atomic‐scale metal centers in carbon frameworks, usually hold the maximum atom utilization, active metal sites, and unique electronic configuration.^[^ ^125^ ^]^ Consequently, they were ordinarily employed in energy conversion and storage because of their catalysis. The unique electron configuration of SACs/carbon with the split energy level and abundant bare metal sites can effectively catalyze the redox reaction of polysulfides.^[^ ^3^, ^36^, ^41^, ^125^, ^126^ ^]^ Generally, in order to gain stable SACs structure and further promote the catalytic properties of SACs/carbon, the transition metal (M) usually interacts with nitrogen groups to form the M–N*_x_* structure.^[^ ^127^ ^]^

**Figure 13:**
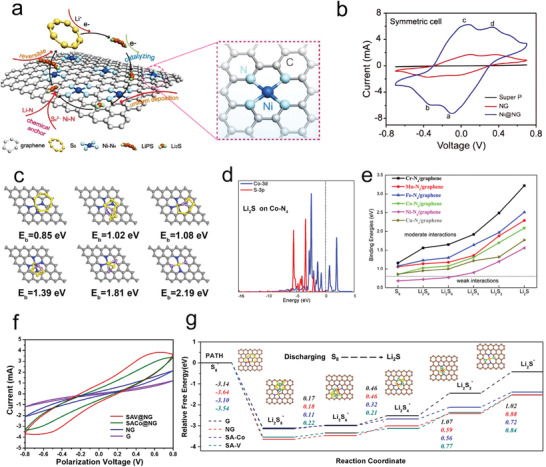
a) The evolution of S_8_⇋Li_2_S on the surfaces of Ni@NG during cycling and b) the symmetric cell tests. a,b) Reproduced with permission.^[^ ^129^ ^]^ Copyright 2019, Wiley‐VCH. c) The optimized structures of S species adsorbed on Co–N_4_/graphene. d) The projected density of states for Li_2_S adsorbed on Co–N_4_/graphene. e) The calculated binding energies of S species on various M–N_4_/graphene. c‐e) Reproduced with permission.^[^ ^15c^ ^]^ Copyright 2018, Elsevier. f) CV curves of symmetric cells based on different electrodes. g) Relative free energy curves for the reduction of polysulfides on different catalysts. f,g) Reproduced with permission.^[^ ^15b^ ^]^ Copyright 2020, American Chemical Society.

Huang and co‐workers proposed an atomic‐scale catalyst Co–N–C to accelerate the polysulfide conversion in a Li—S battery.^[^ ^128^ ^]^ Here, the Co–N–C served as an accelerant to quicken the Li_2_S_1/2_ precipitation dynamics and worked as an atomic adjuster to tune Li_2_S_1/2_ nucleation and reproduction, which resulted in improved discharge capacity. Meanwhile, the atomically diffused lithiophilic and sulfiphilic centers within the conductive substrate completely realized the atomic‐efficient catalytic advantage to enhance the polysulfide conversion. As a result, a low cyclic decay rate (0.10% after 300 cycles), outstanding rate capability (1035 mA h g^−1^ at 2C), and remarkable areal capacity (10.9 mA h cm^−2^ with an S loading of 11.3 mg cm^−2^) were achieved. Besides, single Ni atoms doped N‐graphene (Ni@NG) have possessed immobilization and redox catalysis for polysulfides during the cycling (**Figure** **13**a,b).^[^ ^129^ ^]^ They found that the oxidized Ni sites in Ni–N_4_ structures had reversible catalyzation of polysulfides converting through the formation of S*_x_* ^2−^–Ni–N bonding. Moreover, the DFT calculation revealed that the Cr–N_4_/graphenes exhibited high electroconductivity, temperate binding strength with soluble Li_2_S*_n_* species thanks to the synergistic interaction between metal–S and N–Li atoms, accompanying with a certain quantity of charge transfer between them (Figure 13c–e).^[^ ^15c^ ^]^ Similarly, Zhou et al. recently investigated ten materials (graphene, NG, and NG‐supported SAFe, Mn, Ru, Zn, Co, V, Cu, and Ag) for the potential catalytic conversion toward polysulfides.^[^ ^15b^ ^]^ Importantly, single V catalytic sites show improvement in both the nucleation and decomposition of solid Li_2_S during the charging–discharging processes, which can be ascribed to the low Gibbs energy barrier at the rate‐limiting step (Figure 13f,g). These studies prove the significant role of SACs/carbon in M–S batteries, which can dramatically alleviate the shuttle effects because of their excellent catalytic ability for polysulfides’ redox reaction.

Very recently, Ma and co‐workersdesigned the single‐atom‐Fe and polar Fe_2_N co‐embedded N‐doped graphene (SA‐Fe/Fe_2_N@NG) to catalyze the conversion of polysulfides.^[^ ^130^ ^]^ The single‐atom‐Fe and Fe_2_N served as synergistic sites to expedite the two‐way liquid–solid conversion. The single‐atom‐Fe with plane‐symmetric Fe–N_4_ configuration could selectively catalyze Li_2_S*_n_* reduction. The sulfurophilic Fe_2_N with Fe–N_4_ single‐atom sites assisted not only the obvious reduction of Li_2_S*_n_* to Li_2_S but also excellent catalytic selectivity for Li_2_S oxidation. Besides, Cu SACs with two N and two O atoms coordinated were employed to Na–S battery.^[^ ^131^ ^]^ The hybrid with a large content of Cu single atoms increased the redox kinetics, leading to higher S utilization. Notably, Cu SAC could weaken the S—S bond in S_8_ to generate short‐chain S molecules and avoid the generation of soluble intermediates. Furthermore, Cu SAC offered strong adsorption for Na_2_S_4_ and enhanced Na ions diffusion, which increased the electrochemical reaction kinetics, thus leading to superior S utilization and rate property.

Despite these obvious successes in the SAC‐based cathode, several challenges remain and require further optimization of M–S batteries. 1) The precise construction of doping sites and coordination geometry has not been well achieved. Solutions to these problems are restricted because of the randomness of binding sites in carbon supports. Hence, catalytic materials with homogeneous sites coordinating with heteroatoms are desired. 2) For Li—S chemistry, the affinity toward polysulfides and the Li_2_S nucleation/decomposition behaviors on SACs have not completely been understood up to now, particularly the electron‐donating and withdrawing pathways within SACs and S‐based species during cycling. Thus, it is imperative to figure out the exact mechanisms and redox process via theoretical calculation and in situ characterization. 3) Many of the currently reported SAC‐based catalysts still require further optimization by tuning the atomic metal–N–C structures to enhance the catalytic efficiency and stability simultaneously.

### 5.3 Metal Nanoparticles and Alloys loaded Carbons for Polysulfide Catalysis

**Figure 14:**
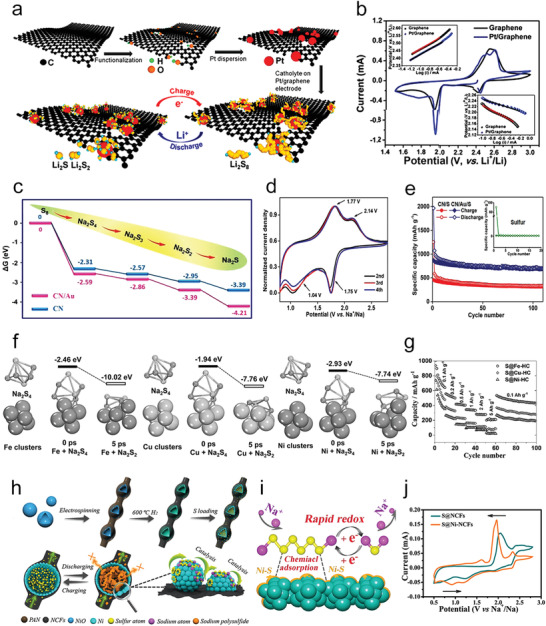
a) Pt electrocatalysts anchored graphene cathodes interacting with polysulfides during the cycling and b) the CV test. a,b) Reproduced with permission.^[^ ^115^ ^]^ Copyright 2015, American Chemical Society. c) Gibbs free energies of different NaPSs binding on different substrates. d) The CV curves of CN/Au/S. e) Cycle capability of different cathodes at 0.1 A g^−1^. c‐e) Reproduced with permission.^[^ ^125d^ ^]^ Copyright 2020, Royal Society of Chemistry. f) The binding energy of Na_2_S_4_ on Fe_6_, Cu_6_, and Ni_6_ nanoclusters. g) Rate performance for various batteries. f,g) Reproduced with permission.^[^ ^135^ ^]^ Copyright 2019, Wiley‐VCH. h) Schematic of the synthesis and working process of S@Ni‐NCFs composite. i) Illustration of polysulfides adsorption on metallic nickel nanoparticles and corresponding catalytic redox reaction. j) Comparison of CV for S@Ni‐NCFs and S@NCFs electrodes. h‐j) Reproduced under the terms of the Creative Commons CC‐BY license.^[^ ^136^ ^]^ Copyright 2019, The Authors. Published by Wiley‐VCH.

Typically, metal nanoparticles and alloys can serve as efficient catalysts in various electrochemical energy conversion systems.^[^ ^21^, ^132^ ^]^ Arava's group first compared the catalytic effect of Pt, Au, and Ni nanoparticles coated Al foil in Li—S batteries and demonstrated the possibility to use metal nanoparticles for catalysis of polysulfides.^[^ ^133^ ^]^ Meanwhile, the Pt nanoparticles loaded graphene have also been studied for the polysulfides redox process through exploring the merits in structure and electrochemistry.^[^ ^115^ ^]^ The hybrid materials can enable a 40% increase in the specific capacity than that of pristine graphene, allowing extra cycling life over 100 cycles, which proved the improvement in polysulfides redox reaction kinetics (**Figure** **14**a,b). Besides, Co nanoparticle‐loaded graphitic carbon has been used for reversibly catalyzing the conversion between soluble high‐order polysulfides and Li_2_S_2_/Li_2_S, resulting in improved reaction kinetics.^[^ ^118^, ^134^ ^]^

Moreover, the high‐efficiency metal‐doped catalysts were also applied in Na–S batteries. Yu and Dou groups have investigated the electrocatalytic effect of gold nanodot‐decorated carbon hosts for advanced Na–S batteries.^[^ ^125d^ ^]^ As presented in Figure 14c–e, the Gibbs free energies of binding between Na polysulfides and N‐doped carbon or Au dispersed N‐doped carbon (CN/Au) have demonstrated that Au nanodots could successfully alleviate the shuttle effect by adsorption from polar interactions, which gave rise to higher discharge voltage and specific capacity. Besides, transition metals combining carbon matrixes have been commonly employed in M–S batteries and achieved wonderful performance.^[^ ^115^, ^137^ ^]^ Qiao group developed hollow carbon nanospheres (HC) with transition‐metal nanoclusters decorated to host S in a Na–S battery.^[^ ^135^ ^]^ Ab initio molecular dynamics simulations revealed that Na_2_S_4_ could effectively decompose into Na_2_S_2_ on these nanoclusters (Figure 14f,g). The results could evaluate the interaction between Na_2_S_4_ and metal nanoclusters; meanwhile, understand the order of the catalytic activities of different metal nanoclusters (Fe > Cu >Ni) according to corresponding batteries’ performance. More exquisitely, carbon fiber‐based concatenated nickel hollow spheres have also been designed to catalyze the conversion kinetics of Na–S batteries; the mobile polysulfides can be anchored through chemisorption by the polar bonds (Figure 14h–j).^[^ ^136^ ^]^

**Figure 15:**
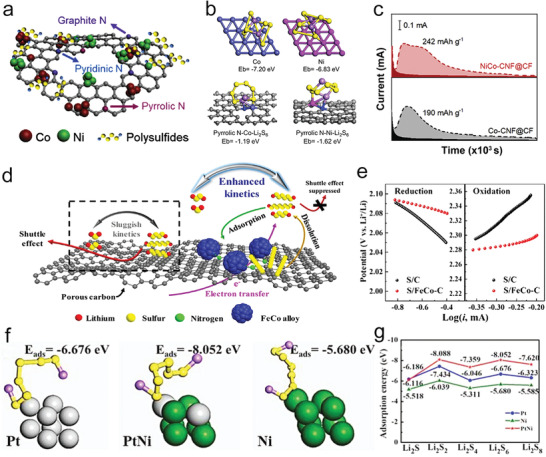
a) The structures and b) adsorption of NiCo‐CNF@CF interlayer toward polysulfides. c) Potentiostatic tests for NiCo‐CNF@CF and Co‐CNF@CF. a‐c) Reproduced with permission.^[^ ^139^ ^]^ Copyright 2019, Elsevier. d) The conversion process of polysulfides on the C and FeCo‐C surface. e) Tafel plots of CV, tested based on S/C and S/FeCo‐C cathodes. d,e) Reproduced with permission.^[^ ^140^ ^]^ Copyright 2020, American Chemical Society. f) Interaction between LiPSs and different catalysts. g) Adsorption energy of various LiPSs on different catalysts. f,g) Reproduced with permission.^[^ ^27^ ^]^ Copyright 2019, Wiley‐VCH.

Alloy nanoparticles with multiple metals can exhibit unique catalytic properties compared to their single metal counterpart.^[^ ^138^ ^]^ The conductive network consisting of CoNi alloy doped carbon nanofibers (NiCo‐CNF) based heterostructure was fabricated elaborately as an interlayer (**Figure** **15**a–c). Notably, the coordination between Ni/Co particles and N–C sites could chemically anchor polysulfides and concurrently accelerated their redox reactions and caused quickly and reversibly electrochemical kinetics in the Li—S battery.^[^ ^139^ ^]^ Recently, the FeCo alloys originated from Prussian blue analogs have been constructed to expedite polysulfide redox reaction and powerfully immobilize polysulfides significantly.^[^ ^140^ ^]^ Herein, the FeCo in situ distributed within the porous carbon (FeCo‐C) uniformly, the close contact of which benefited to interfacial charge transport (Figure 15d,e). The symmetrical cell measurements exhibited that the redox current of FeCo‐C cathode was much higher than pure carbon cathode, indicating faster Li_2_S_6_ redox kinetics on the FeCo‐C cathode. As a result, the as‐developed S@FeCo‐C catalysts achieved superior sulfur utilization and long‐term cycle life. In addition, the Li group proposed a Pt@Ni core–shell bimetallic carbon‐based polysulfide catalyst as an efficient S host (Figure 15f,g).^[^ ^27^ ^]^ The porous carbon frameworks could notably prevent the shuttle of polysulfides through physicochemical confinement while providing effective ion/electron channels. Notably, the Pt@Ni catalyst exhibited superior electrocatalysis performance compared with single Pt or Ni particle, owing to its bifunctional electrocatalytic effect on not only reducing the energy barrier between Li_2_S/Li_2_S_2_, but also quickening the conversion of insoluble products to soluble polysulfide species.

The above reported carbon‐supported metal nanoparticles and alloys have possessed efficient catalysis in M–S batteries. However, metal nanoparticles on carbons usually exhibit insufficient binding ability or undergo reconstruction of chemical compositions, thus resulting in inferior long‐term cycling stability. Moreover, benefiting from the unique electronic and bifunctional redox properties, the transition metal‐based alloys could be promising candidates to achieve high activity and strong stability in M–S batteries compared to their monometallic components.

### 5.4 Metal Oxide Loaded Carbon for Polysulfide Catalysis

**Figure 16:**
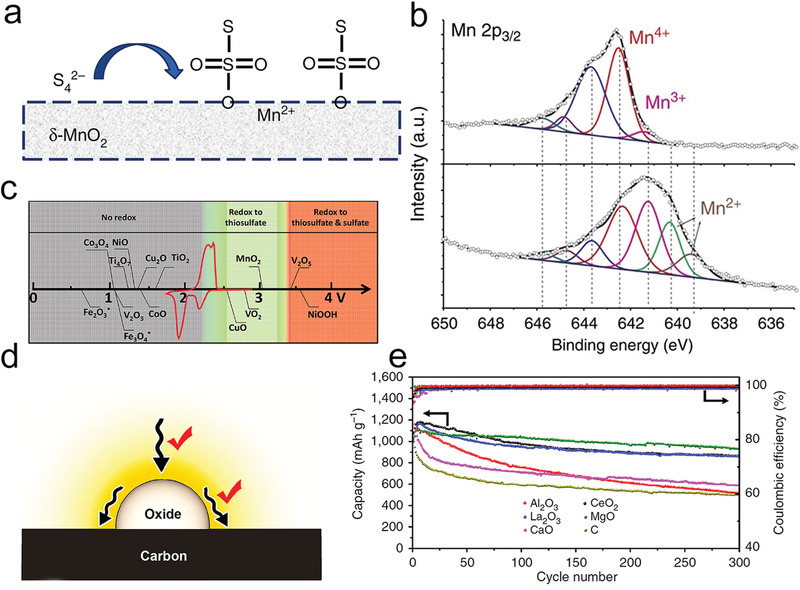
a) Schematic image of initially formed thiosulfate on the *δ*‐MnO_2_ surface. b) Mn 2p_3/2_ XPS of MnO_2_ nanosheets and MnO_2_–Li_2_S_4_. a,b) Reproduced with permission.^[^ ^141^ ^]^ Copyright 2015, Nature Publishing Group. c) The chemical reactivity of diverse metal oxides with polysulfides based on redox potential versus Li/Li^+^. Reproduced with permission.^[^ ^73^ ^]^ Copyright 2016, Wiley‐VCH. d) Mild interaction between polysulfides and nonconductive metal oxides and e) corresponding electrochemical performances. Reproduced with permission.^[^ ^29^ ^]^ Copyright 2016, Nature Publishing Group.

To date, two theories of metal oxides accelerating polysulfide redox have been demonstrated. As mentioned above, one is generating surface‐bound thiosulfate/polythionate intermediates via metal oxides reacting with polysulfides (**Figure** **16**a,b).^[^ ^141^ ^]^ It suggested that metal oxides primarily oxidized the first produced polysulfides into insoluble thiosulfate groups immobilized on their surfaces. Then, the newly generated polysulfides are anchored by the thiosulfate groups through S—S interactions, leading to the generation of polythionate and next transforming into short‐chain Li_2_S. For instance, the transformation of polysulfides followed by thiosulfate formation has been demonstrated on the surfaces of CuO/VO_2_ with anticipated redox potential in the range of 2.4–3.05 V (Figure 16c).^[^ ^73^ ^]^ Normally, the redox potential for generating polysulfides was lower than 2.4 V. Hence, the somewhat higher redox potential of metal oxides might contribute to the oxidation of polysulfides and succeeding thiosulfate formation.

Another theory of catalytic activities highlights the importance of collaboratively helping both the adsorption/diffusion of polysulfides via the moderate polar surfaces of metal oxides.^[^ ^29^ ^]^ It was verified that strong adsorption of polysulfide on a polar surface should be efficient for performance enhancements, while surface diffusion from the nonconductive oxides to conductive carbon substrates is essential to receive electrons.^[^ ^142^ ^]^ For instance, the La_2_O_3_, MgO, and CeO_2_ showed better capacities and stabilities than Al_2_O_3_ in Li—S batteries (Figure 16d,e).^[^ ^29^ ^]^ Even if the binding energy of polysulfides on Al_2_O_3_ surfaces being the largest, the slow surface diffusion reduced the conversion kinetics and deteriorated these problems. Meantime, it was also exposed that the Fe_3_O_4_@C could ensure fast electron/ion transfer and anchor polysulfides in the cathodes through conductive skeletons and mild binding capacity.^[^ ^143^ ^]^ Therefore, moderate bonding with polysulfides and succeeding facile surface diffusion via polar metal oxides can lead to excellent S host materials.

Moreover, Fe_3_C@Fe_3_O_4_@C was utilized as the interfacial coating catalyst on the separator to promote the conversion of lithium polysulfides.^[^ ^144^ ^]^ Based on the prepared materials, the d–p band models have been successfully employed to simulate the Fe‐based polysulfide catalysts for Li—S batteries. The narrower energy bandgap (∆p–d), that is, the average difference within the center of the p‐band and the d‐band in the electron spin upward/downward, corresponds to a reduced reaction impediment and improved rate‐performance of batteries. It is expected that the extension of d–p band theory in catalytic materials could be contributing to future studies in M–S batteries.

The carbon‐supported metal oxides have demonstrated robust chemical interaction with polysulfides and can capture them to relieve the shuttle effects. However, several challenges still exist. 1) The catalytic activities of many reported metal oxides are still not satisfactory. Therefore, exploring new metal oxide‐based catalysts with better performance is still required in the future. 2) In addition, the good electrical conductivity of the matrix to load metal oxides is also vital for highly efficient catalysis, considering that the M–S battery requires an efficient flow of electrons. 3) The hetero atoms doped metal oxides, including N, S, P, and other metals, should also be considered for tuning the electronic structures of the catalysts.

### 5.5 Transition Metal Dichalcogenides Loaded Carbon for Polysulfide Catalysis

Since metallic sulfides have been applied as hydrodesulfurization catalysts to reduce the sulfur content of refined oil production, various metal sulfides were proposed to improve the redox kinetic of M–S batteries. Most researchers concluded that metal sulfides with sulfiphilic sites could strongly interact with polysulfides and possess higher conductivity than metal oxides due to their delocalized electronic microstructures.^[^ ^79^, ^84^, ^145^ ^]^ When TiS_2_, ZrS_2_, and VS_2_ were applied as cathode materials, they could improve accelerated charge transfer and realize mildly bonding toward polysulfides.^[^ ^146^ ^]^ As a result, the redox kinetic of polysulfides could be promoted during the charging–discharging process, thereby resulting in superior rate capabilities.

**Figure 17:**
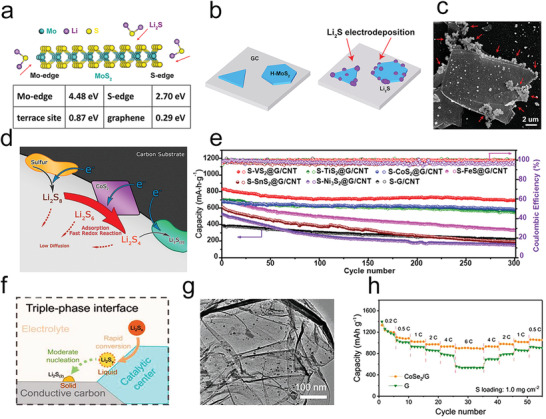
a) Simulations, characterizations, b) schematics, and c) SEM image of Li_2_S deposition onto MoS_2_ and GC substrate. a‐c) Reproduced with permission.^[^ ^149^ ^]^ Copyright 2014, American Chemical Society. d) Polysulfide reduction is accelerated on CoS_2_. Reproduced with permission.^[^ ^7b^ ^]^ Copyright 2015, American Chemical Society. e) Electrochemical performances of various metal sulfides. Reproduced with permission.^[^ ^26^ ^]^ Copyright 2017, National Academy of Sciences. f) Diagram of the polysulfide redox reaction and Li_2_S nucleation. g) TEM of CoSe_2_/G hybrids. h) The rate performance of batteries with a CoSe_2_/G functional separator. f‐h) Reproduced with permission.^[^ ^12b^ ^]^ Copyright 2018, Wiley‐VCH.

A noticeable difference from metal oxides could be the 2D‐layered architectures of metal sulfides comprising two atomic configurations (i.e., basic plane and edge sites). In general, when 2D metal sulfides were employed as catalysts in normal industrial fields, their catalytic activation primarily originated from active edge sites.^[^ ^147^ ^]^ The notable discrepancy among edge sites and basic plane on the catalytic activation uncovered the significance of studying where the reactive activation arises in the 2D metal sulfides. It has been demonstrated that the sulfur could moderately interact with Li^+^ in polysulfides, which weaken reacting energy barriers and quickening redox dynamics.^[^ ^148^ ^]^ The conversion of soluble polysulfides to insoluble S species could also selectively take place along the edge sites of MoS_2_ due to their robust binding energies toward Li_2_S than that of the basal plane (**Figure** **17**a–c).^[^ ^149^ ^]^ The redox kinetic enhancement at edge sites has been further investigated with WS_2_ and MoS_2_; the coordination of unsaturated atoms could improve the charge delivery and interaction with S species.^[^ ^150^ ^]^ From this point of view, a sulfur‐defective MoS_2_ and rGO composite (MoS_2−_ *_x_*/rGO) was prepared to form the accessible Mo sites in the basic plane to achieve excellent catalytic activity of polysulfides.^[^ ^151^ ^]^ Therefore, defect engineering has been studied not only in metal oxides but also in metal sulfides for achieving effective polysulfide confinement, rapid charge transfer, and accessible catalytic sites.^[^ ^152^ ^]^

Aside from 2D‐layered metal sulfides, another formalization of pyrite‐type structures, including CoS_2_, FeS_2_, etc., was also explored.^[^ ^36^, ^153^ ^]^ It has been found that CoS_2_ promoted the polysulfides redox kinetics because of its considerable electroconductivity and excellent sulfiphilic affinity (Figure 17d).^[^ ^7b^ ^]^ As a result, a low capacity decay of 0.034% per cycle at 2.0C and an excellent initial capacity of 1368 mA h g^−1^ at 0.5C was realized via the mechanical mixing of graphene and CoS_2_ microparticles. As mentioned above, the critical characteristics for promoting redox kinetic should be the accelerated charge delivery and appropriate polysulfide binding ability. The Cui group has methodically studied a series of metal sulfides to disclose the related mechanism for catalytically decomposing Li_2_S (Figure 17e).^[^ ^26^ ^]^ The calculation exposed that the order of the magnitude of the Li_2_S dissociation impediment on metal sulfides was Ni_3_S_2_ > FeS > CoS_2_ > SnS_2_ > VS_2_ > TiS_2_, and the dissociation process related to the interaction between S^2−^ in sulfides and the dissociative Li^+^ ions, which may be the key factor for low dissociation barrier compared with conventional carbon hosts.

In general, the electroconductivity of metal selenides is higher than their sulfide counterparts though both are sulfiphilic.^[^ ^153c^ ^]^ Thus, metal selenides show more great potential as catalysts in M–S batteries. Very recently, Yuan et al. prepared a triple‐phase interface among electrolyte/CoSe_2_/G affording synergistic effect of strong chemisorption, high electroconductivity, and superb electrical catalysis, which can promote the kinetic behaviors of soluble polysulfides and regulate the nucleation and growth of insoluble Li_2_S (Figure 17f,g).^[^ ^12b^ ^]^ The uniformly dispersed CoSe_2_ nanodots on rGO nanosheets led to the dense and uniform distribution of sulfiphilic catalytic sites. As shown in Figure 17h, the superior rate performance and capacity recovery demonstrated that the CoSe_2_/G could facilitate sulfur redox chemistry. Likewise, ultrafine Co_3_Se_4_ nanoparticles have been grafted onto the surfaces of the N‐doped 3D carbon matrix.^[^ ^154^ ^]^ The N‐CN‐750@Co_3_Se_4_‐0.1 fulfilled three indispensable requisites for accelerating sulfur conversion reactions, including strong binding energy toward polysulfides, fast transportation for electron/ion, and rapid conversion of polysulfides. Undoubtedly, transition metal selenides possess the superb electrocatalytic capacity for M–S batteries, while utilizing them to immobilize and catalyze polysulfides in M–S batteries is still at its early stage.

To date, the mechanism of these metal sulfides/selenides based electrocatalysis in M–S batteries has not been fully identified. Abundant literature has reported different kinds of metal sulfide‐based catalysts with special composition and morphology, which cause a great prospect in the M–S system because of their unique electronic features, band position, and abundant accessible catalytic sites. The interactions between polysulfide and the active edge sites contribute to the robust trapping effect of metal sulfides/selenides. However, although their adsorbability and catalytic activity hamper shuttling effects have been demonstrated, their underlying mechanisms have not been fully disclosed. In conclusion, much more researches should be proposed to investigating the principle of the catalytic effect of the metal sulfides/selenides concerning the complex redox processes of polysulfides. As such, the understanding of these mechanisms can allow better progress in the future commercialization of M–S batteries.

### 5.6 Metal Nitrides, Phosphides, and Carbides Loaded Carbon for Polysulfide Catalysis

**Figure 18:**
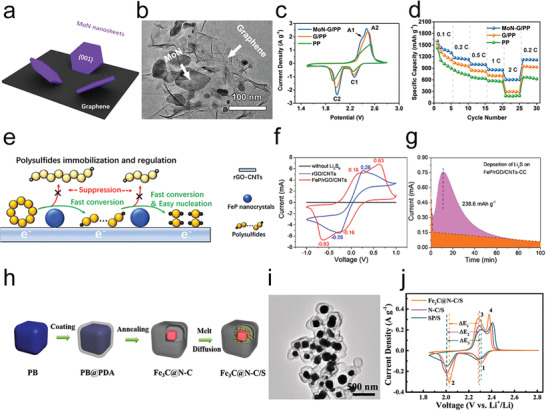
a) Diagram of the structure of the MoN‐G composite. b) TEM images of MoN‐G. c) CV curves of different cells. d) Rate performance of diverse cells. a‐d) Reproduced with permission.^[^ ^156^ ^]^ Copyright 2019, Wiley‐VCH. e) Schematic of FeP nanocrystals immobilizing and regulating polysulfides. f) CV curves of symmetric batteries at 3 mV s^−1^. g) Potentiostatic discharge test of Li_2_S_8_ solution at 2.07 V. e‐g) Reproduced with permission.^[^ ^77b^ ^]^ Copyright 2018, Elsevier. h) Schematic of preparing frogspawn‐like hollow Fe_3_C@N‐C. i) TEM images of Fe_3_C@N‐C. j) CV curves of the Fe_3_C@N‐C/S, N‐C/S, and SP/S electrodes at a scan rate of 0.1 mV s^−1^. h‐j) Reproduced with permission.^[^ ^158^ ^]^ Copyright 2019, Royal Society of Chemistry.

The excellent catalytic activities of metal nitrides originate from their unique electronic structure and high electronic conductivity. As mentioned above, researchers have combined metal nitrides and carbon matrixes to obtain higher catalytic activity and synergistic effect. For instance, Shi et al. in situ introduced niobium nitride onto graphene with ultrafine size and uniform dispersion to fabricate efficient polysulfides impeding layer.^[^ ^155^ ^]^ Here, the graphene could physically hinder polysulfide diffusion and construct highly effective conductive pathways; meanwhile, the polar NbN not only served as chemical reservoirs to capture the diffused polysulfides but also quicken the redox reaction of polysulfides. Besides, the MoN was anchored on graphene sheets to serve as an interlayer, which could immobilize the polysulfides strongly through Mo—S bonding and help the decomposition of Li_2_S (**Figure** **18**a–d).^[^ ^156^ ^]^ In addition to graphene, carbon cloth has been employed to load the VN as polysulfides catalyst owing to its strong chemical adsorption for polysulfides, highly conductive feature (1.67 × 10^6^ Ω^−1^ m^−1^), and even the similar catalytic activity to noble metals.^[^ ^157^ ^]^ Therefore, combining metal nitrides with carbon materials should be a promising strategy to induce new catalytic structures.

To relieve the inherent disadvantages of carbon materials with only physical polysulfide confinement, metal phosphide‐loaded carbons have been investigated as catalysts with excellent electrical conductivity, which could physicochemically anchor the polysulfides and expedite their redox conversion. Yang's group employed the iron phosphide (FeP) nanocrystals to incorporate on the 3D porous rGO‐CNT frame as an effective host for Li—S batteries (Figure 18e–g).^[^ ^77b^ ^]^ Here, the FeP nanocrystals exhibited a very high capability to catalyze the polysulfides conversion and reduce the energy barrier of Li_2_S nucleation, thereby leading to high rate capability and stability. Considering the catalytic effects of FeP for polysulfides, the FeP@C nanotube arrays uniformly dispersed carbon cloth fiber has been fabricated.^[^ ^159^ ^]^ The energy center of the p band in FeP was drastically moved to the Fermi level, decreasing the energy gap between bonding and antibonding, finally resulting in the promotion of electron transfer and polysulfides transformation dynamics. Similarly, the cobalt phosphide surface's oxidation layer could also bind polysulfide by Co—S bonding chemically.^[^ ^157^ ^]^ It was demonstrated that the oxidation‐activated polysulfide binding mechanism on the surface of catalysts could be a universal phenomenon because many substances, such as Ni_2_P, FeP, MoP, CoS, and CoSe_2_, had the ability to anchor polysulfide with oxide layers.

Metal carbides have excellent performance in electrocatalysis.^[^ ^160^ ^]^ Very recently, Li's group reported polyoxometalate (POM) derived hierarchical carbonaceous support with a uniform dispersion of ultrasmall *α*‐MoC_1−_ *_x_* nanoparticles,^[^ ^161^ ^]^ which not only had a strong surface attraction for polysulfides but could significantly promote their conversion speed. MoC_1−_ *_x_*/C could promote the reduction of Li_2_S_4_ to insoluble Li_2_S_2_ or Li_2_S, which contained the heterogeneous nucleation and development of the solid phase. Thus, they obtained good cycling stability after 200 cycles at 1600 mA g^−1^. The structures of batrachian eggs in nature ceaselessly inspired researchers; for example, we have reported bufo‐spawn shape open‐mesoporous N‐doped‐carbon nanofibers with atomic Fe–N*_x_* (OM–NCNF–FeN*_x_*) as innovative oxygen cathode for Mg–air batteries.^[^ ^162^ ^]^ Similarly, a frogspawn‐like hollow Fe_3_C@N‐C was prepared to serve as a highly efficient cathode for high‐rate Li—S batteries (Figure 18h,i).^[^ ^158^ ^]^ The coupling effect from N‐doped carbon and polar Fe_3_C promoted polysulfide conversion kinetics and adsorption ability to polysulfides, leading to the smaller potential between the reduction and oxidation current peaks (Figure 18j), which suggested a quick redox reaction of polysulfides.

Metal phosphides display outstanding stability, making them appropriate for energy storage applications. P atoms with higher electronegativity can draw electrons from metal atoms and serve as a base to capture positively charged species. Meanwhile, the metal phosphides have moderate adsorbability for Li_2_S_6_, which endows them as promising candidates for cathode materials in M–S batteries except for their difficult preparation process. Besides, the metal nitrides are also promising cathode materials, which can facilitate the transport of ions and electrons with good conductivity, thus resulting in the fast conversion of the adsorbed sulfur species on them. However, nitrides have the strongest adsorbability.^[^ ^23^ ^]^ The adsorbability for Li_2_S_6_ on nitrides surface is so strong, which can break the Li—S bonds in LiPSs and thus lead to partial surface sulfurization on metal nitrides. Moreover, according to the Sabatier principle, too strong binding could block the surface reaction sites to lower the catalytic reaction. So, it is a good strategy to slightly lower the interaction between polysulfides and metal nitrides by surface doping of C, P, and S.

## 6 Composition Design Principles of Polysulfides Catalysts

To optimize M–S batteries performances, promising structures and catalytic centers have been desired in recent literature. Currently, the deadly shuttle effect and sluggish polysulfides’ redox kinetics can lead to insufficient electrical performances of M–S batteries.^[^ ^41a^ ^]^ Varieties of catalytic materials, including organic‐, metal‐, and carbon‐based materials, have been applied to promote batteries' performance. The respective weakness and strengths of different materials motivated us to explore the adsorbing, diffusing, and converting of polysulfides on catalysts. Along with this idea, we can design and manufacture a smooth polysulfide adsorbing and converting process by improving the polysulfide confining capability and electrical conductivity of polysulfide catalysts. Many efforts have been contributed to impelling redox kinetics by designing delicate nanostructures and catalytic centers. Thus, this section will first discuss the general structural design principles on effective polysulfide catalytic strategies by increasing sulfur redox kinetics.

Most conducting polymers own good electron conductivity to be directly employed as sulfur hosts for M–S batteries. Moreover, N‐containing groups, especially amino groups with a positive charge on the backbones of conducting polymers, can act as active sites to adsorb polysulfide anions with a negative charge through electrostatic interaction and afford a rapid redox reaction.^[^ ^33^ ^]^ Besides the conducting polymers, the N‐doped COFs and MOFs with a porous structure and high specific surface have also revealed application potentials for polysulfide catalytic conversion. However, these polymers' insufficient electrical conductivity may obstruct the electron transfer at the catalytic sites, thus hindering the fast polysulfide conversion. Further integrating the COFs and MOFs with conductive substrates is highly needed to increase the catalytic activity and stability.

N‐doped carbons have already been widely used as electrocatalysts in many catalytic applications; recent studies have displayed that they can also be used for polysulfide catalysis.^[^ ^13^, ^41^ ^]^ Besides the metal‐free heteroatoms doped carbon, the metal–N*_x_* doped carbons have also demonstrated good polysulfide catalytic activities.^[^ ^15b^ ^]^ Polar inorganic metal is another efficient type of polysulfide catalytic material. It has been intensively studied recently due to its strong physical or chemical interactions between transition metal cations and polysulfides, including Lewis acid–base interactions and M–S bonds forming.^[^ ^7^, ^106^, ^141^, ^148^, ^163^ ^]^ For instance, recent theoretical studies revealed that metal oxides could efficiently adsorb polysulfides by transition metal oxide–sulfur interactions.^[^ ^73^ ^]^ Besides, Tao et al.^[^ ^29^ ^]^ used theoretical calculations of Li^+^ ion diffusion energy and binding energy to explore the catalytic behavior of a series of metal oxides (CeO_2_, Al_2_O_3_, La_2_O_3_, MgO, and CaO). They found that balancing the adsorption and diffusion of polysulfides on the surface of catalysts is conducive to maintaining the activity of sulfur species during the cycle, thereby improving battery performance.

The sulfuration and phosphorization of metal oxides can further boost the catalytic activities of polysulfides. After phosphorization, the p band center in metal phosphides had a distinct upshift toward Fermi level compared with that in metal oxides, which promoted the interfacial electron transfer dynamics.^[^ ^23^, ^159^ ^]^ By calculating the binding energies of polysulfides with CoP, CoS_2_, Co_3_O_4_, Co_4_N, it has been found that the CoP had moderate binding energies for Li_2_S_6_ and Li_2_S, which exhibited the fastest Li_2_S diffusion kinetics and the best battery performance.^[^ ^23^ ^]^ Therefore, the gentle adsorption and diffusion of polysulfides on the catalyst surfaces helped promote the conversion kinetics of polysulfides and could be the design direction of future polysulfide catalysts.

Overall, the prerequisites for an ideal catalyst in the M–S battery involve favorable electrical conductivity, enough exposure to active surfaces and sites, moderate affinity toward polysulfides, and accelerated redox kinetics. Thus, they can easily anchor polysulfide and transfer electrons rapidly, the process of which is the essence of electrochemical catalysis. As for Li—S batteries, the main factor of the shuttle effect may originate from the high *E*_a_ during Li_2_S_6_→Li_2_S_4_ and Li_2_S_4_→Li_2_S_2_/Li_2_S, which leads to the accumulation of soluble polysulfide in the electrolyte, and thus the shuttle effect occurs. Therefore, designing suitable electrocatalysts to lower this barrier and accelerate the conversion rate of Li_2_S_6_→Li_2_S_4_ and Li_2_S_4_→Li_2_S_2_/Li_2_S may be a fundamental solution.

Since this paper mainly discusses the catalytic transformation of polysulfide in the process of charge and discharge, the materials discussed in this paper are mainly cathode materials with catalytic effects. We have compared the effects of materials on battery performance based on their catalytic centers. In terms of material design, the material's catalytic capacity is generally judged according to the capacity decay of the battery and the binding energy between the material and polysulfide. In general, the higher the material's catalytic efficiency, the higher the utilization of sulfur, and the lower the capacity decay of the batteries.

In **Table** **1**, we have compared the influence of various catalytic materials on the capacity decay of the battery, in which the capacity decay from low to high is as follows: single atom doped carbon materials ≈ metal compounds doped carbon materials < carbon‐free inorganic materials ≈ organic framework materials. It can be indicated that the single‐atom and metal compound doped carbon material possesses superior catalytic conversion efficiency for polysulfides. Besides, we also compared the adsorption energies between various catalytic materials and polysulfide compounds in **Table** **2**. Among them, the binding energy between single atom/metal compounds doped carbon materials and polysulfide is relatively mild, and this mild binding energy combining with effective electron transport between them could significantly promote the redox reaction kinetics of metal–sulfur batteries.

**Table 1:** Performance comparison of different catalytic materials for Li—S batteries

Materials		Catalytic centers	Capacity retention [%, for 100 cycles]		C rate	Sulfur loading [mg cm^−2^]	References
			Without catalyst	With catalyst			
Conducting Polymer	NPGO	—NH^+^ ═/—N═	64.1	72.9	1C	1.4	[33]
COF	CTP‐1	Perylene	80	81.3	1C	1.3	[14b]
	TP‐BPY‐COF	Pyridine units	50	71	0.2C	–	[164]
MOF	Ni‐ZIF‐8@CC	Ni species	–	≈82	0.13C	5.5	[16]
	F‐Cu‐BTC‐PMIA	Cu‐BTC	61.7	62.9	0.5C	2.1	[165]
	Ce‐MOF‐2	Ce(IV)‐cluster	87.5	93.9	1C	2.5	[14c]
	Na_2_Fe[Fe(CN)_6_)]	Fe(CN)_6_	–	83.3	2C	–	[166]
Inorganics	Fe(0.1)/Co_3_O_4_	Fe, vacancy	47.6	78.9	0.2C	–	[70a]
	Co_3_S_4_	Co_3_S_4_	–	85.1	5C	≈4	[79]
	ZnS	ZnS	–	75	0.2C	7.1	[68b]
	SnS_2_	SnS_2_	57.1	84.6	0.2C	3.1	[78]
	Co_4_N	Co, N	–	87.5	2C	1.5–2	[75]
	Ni_3_FeN	Fe, vacancy	–	92	0.5C	4.8	[68a]
	TiN‐S	Ti—S, Ti—O, Ti–N	94	97	1C	3.4	[68c]
	VN‐NBs	VN	–	94.7	2C	1.2	[72]
Single atoms/C	SAV@NG.	SAV	77.5	91.8	0.5C	5	[15b]
	SAFe@gC_3_N_4_	SAFe	–	96.6	2C	2.3	167]
	Co−N/G	Co−N	77.4	89.5	1C	2	[36a]
	Ni@NG	Ni	–	93.4	1C	6	[129]
	Zn_1_‐HNC	Zn	63	98	1C	1.5	[168]
	Fe/C_2_N	Fe	–	92.3	3C	3	[169]
Metal compound/C	MOF‐Co_4_N	Co_4_N	72	94.5	1C	1	[170]
	MVN@C NWs	VN	62	76.2	1C	2.8	[171]
	MoN‐NC	MoN	88.6	93.1	0.5C	1.5	172]
	h‐Co_4_N@NC	Co_4_N	75	93.6	0.1C	1.5	[173]
	Ni_12_P_5_/CNTs	Ni_12_P_5_	82.8	97	0.5C	3	174]
	CNT‐MoP	MoP	85.5	91.5	1C	1	[175]
	CoP@HPCN	CoP	–	93.3	0.2C	2.3	[24]
	FeP/rGO/CNTs	FeP	87.5	97.5	1C	1	[77b]
	CC@CoP/C‐S	CoP	89.6	94.5	1C	1.81	[176]
	Ni_2_P@NPC	Ni_2_P	80	99	0.2C	3.4	[177]
	MoS_2−_ *_x_*/rGO	MoS_2−_ *_x_*	52	76.3	0.5C	1.5	[151]
	MoS_2_@HCS	MoS_2_	45	87.5	0.5C	–	[148]
	CP@NCNT@CoS_3_	CoS_3_	66.7	82	0.13C	6	178]
	ZnS@NC	ZnS	24	74.1	0.2C	2	[36b]
	MoSe_2_@rGO	MoSe_2_	60	69.2	1C	1.22	[179]
	Fe_1−_ *_x_* S‐NC	Fe_1−_ *_x_* S	63.2	99	0.5C	8.14	[180]
	CS@HPP	CoSe	81.8	96.6	1C	3.7	[107b]
	Fe_3_O_4_@C	Fe_3_O_4_	73.7	99.5	1C	2.85	143]
	TiC@G	TiC	60	70	0.2C	3.5	[37]
	N‐CN‐750@Co_3_Se_4_	Co_3_Se_4_	–	77.2	0.2C	3.1	[154]
	V_2_O_3_@C	V_2_O_3_	–	88.8	1C	3.7	[6b]
	Fe_3−_ *_x_* C@C	Fe_3−_ *_x_* C	69	77.7	1C	1.2	[106a]
	A‐Nb_2_O_5−_ *_x_* @MCS	Nb_2_O_5−_ *_x_*	70.2	93.3	1C	5.8	[181]

NPGO: N, P‐containing polymer coated on GO; CTP: conjugated triazine‐based polymers; MPc‐COFs (M = Ti, V, Cu): transition metal phthalocyanine COFs; Ni‐ZIF‐8@CC: nickel‐doped ZIF‐8 deposited on carbon cloth; TiN‐S: Ti—O bonds in the surface oxidation layer of Ti–N were partially replaced by Ti—S bonds in H_2_S atmosphere; VN‐NBs: vanadium nitride nanobubbles; SAV@NG: vanadium single atoms on N‐doped graphene; Ni@NG: Single nickel (Ni) atoms on nitrogen‐doped graphene; Zn_1_‐HNC: single atom Zn modified hollow carbon sphere nanoreactor; MVN@C NWs: conductive mesoporous VN nanowires encapsulated with conductive C; h‐Co_4_N@NC: Co_4_N nanoparticles in N‐doped carbon, which forms the outer shell of a double‐shelled hollow nanocage; CoP@HPCN: CoP nanoparticles confined in hollow polyhedra/ carbon nanotube; CC@CoP/C‐S: integrating well‐conductive carbon cloth (CC), well‐defined carbon‐encapsulated CoP nanosheets arrays (CoP/C), and the loaded electroactive sulfur; CF/FeP@C: FeP@C nanotube arrays vertically deposited on carbon cloth fiber; Ni_2_P@NPC: Ni_2_P decorated with N and P‐doped carbon nanoflakes; CP@NCNT@CoS_3_: CoS_3_ anchored on the surface of the nitrogen‐doped carbon nanotubes (NCNTs) growing on carbon paper; CS@HPP: CoSe electrocatalyst with hierarchical porous polyhedron; NC/MoS_3_ NBs: hollow‐amorphous N‐doped carbon/MoS_3_ nanoboxes; A‐Nb_2_O_5−_ *_x_* @MCS: amorphous, and oxygen deficient niobium pentoxide nanocluster embedded in microporous carbon nanospheres.

**Table 2:** Binding energies between sulfur species and catalytic materials

Catalytic materials		S_8_	Li_2_S_8_	Li_2_S_6_	Li_2_S_4_	Li_2_S_2_	Li_2_S	References
COF	CTP‐1	–	0.72	0.78	0.8	1.11	1.21	[14b]
	TiPc‐COF	–	5.99	7.29	4.43	–	5.62	[60]
	VPc‐COF	–	5.1	4.98	4.36	–	5.62	[60]
	CuPc‐COF	–	1.3	1.32	1.38	–	2.83	[60]
MOF	Ni‐ZIF‐8@CC	–	3.35	3.81	3.54	–	–	[16]
	Ce‐MOF‐2	–	–	2.48	2.78	2.35	–	[14c]
	Mn‐HAB‐CP	0.78	2.32	1.63	1.8	2.27	2.6	[182]
	[Ni_6_(BTB)_4_(BP)_3_]	–	6.02	5.34	5.06	3.52	–	[183]
	Cu‐BHT	0.678	1.682	1.588	1.824	2.622	3.122	[67b]
Inorganics	Co_3_S_4_	–	1.68	1.61	2.26	–	–	[79]
	TiN	1.19	4.02	2.79	4.87	4.58	4.28	[68c]
	TiN‐S	1.05	4.87	1.75	7.12	6.61	7.19	[68c]
	VN	2.21	3.46	3.01	3.27	3.14	3.86	[72]
	Black phosphorus	–	1.86	–	2.27	2.33	3.05	[94a]
	phosphorene	–	–	0.92	0.94	1.94	2.5	[94b]
Single atoms/C	Cr–N_4_/graphene	1.18	1.58	1.62	1.89	2.49	3.25	[15c]
	Mn–N_4_/graphene	1.11	1.15	1.17	1.38	1.82	2.26	[15c]
	Fe–N_4_/graphene	1.11	1.23	1.29	1.62	1.95	2.55	[15c]
	Co–N_4_/graphene	0.81	1.02	1.09	1.29	1.68	2.1	[15c]
	Ni@NG	0.96	1.82	2.69	2.16	4.19	5.11	[129]
	Fe/C_2_N	8.82	9.92	8.98	7.02	6.75	5.88	[169]
Metal compound/C	MoN‐NC	1.43	2.04	1.89	1.73	2.22	2.74	[172
	CoP@HPCN	–	–	5	1.46	3.3	5.41	[24]
	FeP/rGO/CNTs	3.37	2.83	3.18	2.8	2.42	1.98	[77b]
	CC@CoP/C‐S	1.17	2.42	1.87	2.22	3.07	3.57	[176]
	CF/FeP@C	0.13	3.05	2.63	2.75	4.09	4.1	[184]
	MoS_2_@HCS	0.06	0.1	0.21	0.31	0.66	0.88	[148]
	ZnS@NC	0.71	2.23	2.61	2.24	2.51	3.22	[36b]
	CS@HPP	7.52	8.1	10.06	7.68	7.75	8.83	[107b]
	V_2_O_3_@C	5.828	7.579	6.52	6.625	7.928	5.287	[6b]
	NC/MoS_3_ NBs	1.65	2.71	2.68	2.95	4.82	5.81	[107a]

## 7 Polysulfide Catalyst under Extreme Conditions

As mentioned in our previous mechanism section, in the discharge process of Li—S batteries, about 3/4 of the capacity comes from the catalytic process of soluble Li_2_S_4_ to insoluble Li_2_S.^[^ ^185^ ^]^ While being reduced to Li_2_S, the polysulfides are adapted to deposit on the surface of the cathode uncontrollably, thus generating large particles. Its poor electronic conductivity and high activation energy make it difficult to reuse in subsequent cycles. Furthermore, these problems will deteriorate under the conditions of high loading and lean electrolyte. Obviously, it requires electrochemical catalysts to solve this issue during discharge/charge processes. Therefore, the research on materials with highly active catalytic sites under such harsh conditions is very meaningful. To achieve high energy density and high practicability, a series of practical parameters such as electrolyte/sulfur (E/S) and sulfur loading ratios are supposed to be restricted. It is still a critical challenge to maintain high sulfur utilization and stable cyclability in a battery device featuring high sulfur loading (above 5 mg cm^−2^) and low E/S ratios of 3–11 µL mg^−1^.^[^ ^186^ ^]^

For catalysts used in high loading Li—S batteries, first, it is necessary to have a strong adsorption effect on a large number of polysulfides caused by high loading or physical entrapment of polysulfides. Recently, the Cui group proposed a “high‐tortuosity and high‐sulfur‐philicity” mechanism whereby large electrode tortuosity and high oxygen concentration were the crucial parameters for controlling the diffusion behaviors of soluble active materials inside the cathode, which was significant for enhancing the electrochemical properties and cycling lives of ultrahigh‐loading S electrodes. Benefiting from the high‐tortuosity and high‐sulfur‐philicity features, a high areal capacity of 21 mA h cm^−2^, ≈60% capacity hold at rapid charging rate (16 mA cm^−2^), and 98.1% retention with steady cathodic resistance after 160 cycles were achieved.^[^ ^187^ ^]^

Then, a large number of catalytic sites are needed to quicken the conversion kinetics of polysulfides. Based on a large number of current reports, transition metal compounds are increasingly used in high‐load Li—S batteries due to their robust interaction with polysulfides, including oxides,^[^ ^188^ ^]^ sulfides,^[^ ^189^ ^]^ nitrides,^[^ ^68c^ ^]^ and phosphides.^[^ ^190^ ^]^ For example, the reported 3D graphene/1T MoS_2_ aerogel was used as the cathode material in the Li—S battery. It maintained a good performance at a sulfur content of 10 mg cm^−2^ with the 1181 mA h g^−1^ capacity at 0.1C and a capacity decay rate of 0.08% per cycle after 500 cycles at 1C.^[^ ^191^ ^]^ As for transition metal nitrides, the introduction of a sulfur doping strategy to restore the catalytic activity of nano‐TiN significantly promoted the rapid conversion of polysulfides.^[^ ^68c^ ^]^

Finally, during the charging process, the insoluble and nonconductive Li_2_S is oxidized to soluble polysulfides and further oxidized to solid sulfur. This process involves a larger energy barrier, especially in high loading Li—S batteries, a large amount of Li_2_S will deposit on the cathode, and the reusability of Li_2_S is essential for battery performance. Therefore, introducing catalytic sites on the conductive substrate to accelerate the Li_2_S conversion process is also an important method to improve the performance of high‐load Li—S batteries. For example, MoP nanoparticles played an effective role in Li—S batteries under low E/S rate conditions.^[^ ^190^ ^]^ MoP nanoparticles facilitated the reversible electrochemical conversion reactions of sulfur and thus promoted the deposition of solid sulfur species on the electrode uniformly without electrochemically isolated aggregates. Furthermore, it is also found that the MoP‐assisted polysulfide catalytic conversion can help avoid the shuttle effects, thus maintaining a smooth and clean surface of Li metal anode.

For Li—S batteries with lean electrolyte applications, we need to consider the synergy between the electrode and the electrolyte. Thus, the designed catalyst needs to have the following characteristics: moderate porosity for easy electrolyte penetration and high ionic conductivity, adequate specific surface area for sufficient interface reaction, and fast sulfur redox reaction. The decrease of electrolyte may lead to a partially wetted electrode surface, which will lead to an insufficient sulfur conversion reaction. A “tube on cube” material with a 3D structural connection has been developed to solve the above issue. The corresponding battery could achieve excellent rate performance up to 10C and long cycling performance over 2000 cycles at 9.2 mg cm^−2^ sulfur loading and poor electrolyte (E/S = 6 µL mg^−1^).^[^ ^192^ ^]^ Moreover, it is also proved that low E/S would cause high polysulfide concentration and high electrolyte viscosity, leading to severe blockage of ion transport and increased electrochemical impedance.^[^ ^193^ ^]^ More importantly, the deposition of Li_2_S becomes very slow, which limits the rate capability of Li—S batteries. Therefore, catalytic Li_2_S deposition kinetics at high polysulfides concentration is also an important strategy to improve Li—S batteries. Of course, considering the industrial production of Li—S batteries, it may also involve quality control of lithium anode. However, it has little influence on polysulfide catalytic conversion, so the structural design of lithium anode is not discussed in this review.

## 8 Conclusion and Perspectives

In this timely review, we summarize and comment on the most recent advances in designing polysulfide catalytic materials for fast‐kinetic and long‐life M–S batteries. The currently reported chemistry and mechanism for the catalytic conversion of polysulfides have been summarized in detail. The rational design of diverse polysulfide catalytic materials to accelerate the redox kinetics in M–S batteries is comprehensively discussed to solve the common failure of M–S batteries, i.e., shuttle effect, from performance to the mechanism. As a newly emerging and rapidly developing research area, the catalysts for polysulfides’ redox reactions have obtained significantly increased achievements recently. While compared with the sharp development of other electrocatalytic fields, the investigations on such electrochemical catalysts for accelerating pSRR and pSOR still lie in the very beginning stages and leave a large number of unknowns.

Although the corresponding investigation is still in an infant stage, benefiting from their unique structure, multifunctional feature, and effective catalytic performances, the polysulfide catalytic materials have a tremendous attraction and present promising prospects in future M–S batteries. It is noted that some challenges and practical outlooks would need to be carefully considered in future investigations on the different polysulfides catalytic materials. Especially for the reasonable design of high‐performance catalysts, many challenges and vital scientific issues are pressing to be discussed and dealt with when they are involved in future high‐capacity and quick‐charging M–S batteries, which are urgently needed in the next‐generation energy storage systems. Some key points discussed in our review are summarized below, which remain as a focus for the further development of M–S batteries.

1. Insufficient understanding of the mechanisms of polysulfide catalytic conversion in M–S batteries.
  - The future design of polysulfide catalysts should be guided by the principle understanding of detailed parameters connected with their catalytic activities. The capacity for binding with polysulfides, the diffusivity of M^+^ (M = Li, Na, K), deposition and dissolution of M_2_S, and the defects and coordination atoms of metal centers in catalysts should be considered systematically when exploring the corresponding mechanisms.
  - The interaction strength between catalysts and polysulfides affects the effectiveness of sulfur cathodes. The stronger the interaction, the stronger the bonding. However, a too robust bonding will negatively affect the cycling conversion of sulfur species and thereby capacity loss. A deeper insight into the mechanisms will contribute to designing more effective catalysts, thus reducing the catalyst content in sulfur electrodes.
  - Most researchers believe that the performance degradation is related to electrode pulverization or shuttling effect without considering the deterioration of the catalysis and adsorption capability of catalysts. The understanding and in situ evaluation of polysulfide catalysts are required, including exploring the dynamic interaction mechanisms between catalysts and polysulfides.
2. Barriers to organic frameworks as polysulfide catalysts.
  - Although many recent studies have demonstrated that pristine COFs and MOFs can be directly employed for polysulfide catalysts, the surfaces of these organic frameworks could suffer an irreversible phase transition during cycling, leading to changes of catalytic sites. Therefore, the actual catalytic sites on the surface should be confirmed by more detailed testing.
  - The present COFs/MOF‐derived catalysts are primarily generated in the laboratory with well‐controlled conditions. Scaling up the commercial production needs better process control at low cost and high reproducibility.
3. Lack of in‐depth investigation of single‐atom catalytic activity.
  - Various SACs/carbon catalysts have been employed in M–S batteries. However, few investigations have been represented to confirm the influences of electronic configurations of SACs on redox kinetics of M–S batteries. Notably, the activities of single atoms depend heavily on their electronic structures; in the meantime, this electronic structure can be adjusted by adjacent coordinated atoms and dual‐metal sites.
  - The current mass loading of SACs is very low, which inhibits the catalytic efficiencies of polysulfides. Exploration of suitable and efficient methods to synthesize SACs with high density and good stability is essential for future advanced M–S batteries.
4. Challenges of catalyst design under extreme conditions
  - The high porosity of catalysts should be a double‐edged sword in M–S batteries because higher porosity requires a higher E/S ratio to keep the unaltered electrochemical stability of high‐sulfur‐loading M–S batteries. In this case, balancing the porosity of the catalyst is a key challenge in creating fast ion transport channels while reducing electrolyte consumption.
  - In the commercialization process of M–S batteries, it has been pointed that the areal S content larger than 4 mg cm^−2^ and an E/S ratio lower than 3.0 µL mg^−1^ are the preconditions to realize the energy density of 500 Wh kg^−1^. In this case, the design and evaluation of polysulfide catalysts under these extreme conditions should be paid more attention to the future research.
5. Challenges in designing Na–S and K–S batteries.
  - In K–S or Na–S batteries, electrode damage is a severe concern. K–S has a 296% expansion from S_8_ to K_2_S, and Na–S has a 170% expansion from S_8_ to Na_2_S. Therefore, designing porous materials or carbon frameworks with improved elasticity and flexibility is required. Recent studies have shown that the introduction of reinforcing phases into the cathode, such as carbon nanofibers, provides great promise to tolerate massive volume expansion.^[^ ^39^, ^194^ ^]^
  - The KPSs and NaPSs may have different reactive properties compared to the LiPSs; thus, more in situ and theoretical investigations of polysulfide catalytic conversion in K–S and Na–S should be carried out and compared with Li—S, especially the diffusivity, deposition, and dissolution of NaS*_x_* and KS*_x_*.

In general, the pSRR and pSOR mechanisms and the corresponding electrochemical catalysts concluded in this review have provided a new direction for design future high‐performance M–S batteries. We have illustrated the most recent advances in the rational design of polysulfide catalytic materials to accelerate the kinetics in M–S batteries during charging–discharging processes. The performances of M–S batteries are expected to be further enhanced by designing and fabricating more highly efficient catalysts for the challenge from high loading mass and lean electrolytes. It is believed that this progress review will provide a cutting‐edge understanding of different engineering categories of polysulfide catalysts, and thereby offering both experimental and theoretical guidance for optimizing future high‐performance M–S batteries and many other related battery systems.

## Conflict of Interest

The authors declare no conflict of interest.

## Author Contributions

M.C. and R.Y. contributed equally to this work. M.C., R.Y., S.L., W.Y., and C.C. conceived the idea and structure of the manuscript. M.C., R.Y., Z.Y., X.T., T.M., F.R., and C.C. wrote the manuscript. M.C., R.Y., S.C., S.L., W.Y., and C.C. designed and prepared the images in the manuscript. S.L., W.Y., and C.C. wrote and corrected the manuscript.
